# E3 ligase TRIM8 suppresses lung cancer metastasis by targeting MYOF degradation through K48-linked polyubiquitination

**DOI:** 10.1038/s41419-025-07421-6

**Published:** 2025-02-11

**Authors:** Chi-Hsuan Wei, Chia-Wei Weng, Chih-Ying Wu, Hsuan-Yu Chen, Ya-Hsuan Chang, Gee-Chen Chang, Jeremy J. W. Chen

**Affiliations:** 1 Graduate Institute of Biomedical Sciences, National Chung Hsing University, Taichung, Taiwan; 2 School of Medicine and Institute of Medicine, Chung Shan Medical University, Taichung, Taiwan; 3 Department of Pathology and Laboratory Medicine, Taichung Veterans General Hospital, Taichung, Taiwan; 4 Institute of Statistical Science, Academia Sinica, Taipei, Taiwan; 5 Institute of Molecular and Genomic Medicine, National Health Research Institutes, Miaoli, Taiwan; 6 Division of Pulmonary Medicine, Department of Internal Medicine, Chung Shan Medical University Hospital, Taichung, Taiwan; 7 Graduate Institute of Molecular Biology, National Chung Hsing University, Taichung, Taiwan

**Keywords:** Non-small-cell lung cancer, Metastasis, Tumour biomarkers, Tumour-suppressor proteins, Oncogenes

## Abstract

Ubiquitination is a posttranslational modification that regulates tumour progression-associated proteins through the ubiquitin‒proteasome system, making E3 ligases potential antitumour targets. Here, we report that TRIM8, a member of the TRIM family and an E3 ligase, can act as a tumour suppressor in non-small cell lung cancer (NSCLC). Both gain- and loss-of-function experiments revealed that TRIM8 inhibits the proliferation, colony formation, migration and invasion of NSCLC cells. Experiments with a xenograft model showed that TRIM8 expression suppresses tumour metastasis in vivo. Moreover, low expression of TRIM8 was associated with poor overall survival in both the Taiwanese and GEO lung cancer cohorts. TRIM8 overexpression in lung cancer cells reduced MYOF expression, and restoring MYOF rescued cell migration in TRIM8-overexpressing cells. TRIM8 targeted MYOF for K48-linked ubiquitination, facilitating proteasome-mediated degradation and subsequently suppressing the extracellular secretion of MMPs. Our results provide new insights into the contribution of TRIM8 to lung cancer progression, suggesting that TRIM8 is a new biomarker and a novel therapeutic target for lung cancer.

## Introduction

Lung cancer is the leading cause of malignancy-related mortality worldwide, including in Taiwan. Based on histological features, NSCLC can be divided into lung adenocarcinoma, squamous cell carcinoma and large cell carcinoma [1]. Although progress has been made in therapeutic strategies, including surgery, radiation, chemotherapy, and targeted therapies, delays in diagnosis or widespread metastasis contribute to a poor prognosis [2].

The lung tumour microenvironment (TME) plays an important regulatory role in tumour growth and metastasis [3]. The lung TME includes innate and adaptive immune cells with tumour-promoting or tumour-suppressing functions [4]. Macrophages are recruited to the TME and become tumour-associated macrophages, a dominant immune cell type in the TME [5]. Of the two main macrophage subpopulations, M1 macrophages, which induce a proinflammatory response to protect the body from injury, are considered antitumour macrophages [6]. In contrast to M1 macrophages, M2 macrophages, which participate in angiogenesis, wound healing and tissue remodelling, are immunosuppressive and promote tumour progression [5]. In our previous report, we showed that M1 macrophage coculture decreases the viability and proliferation of A549 lung cancer cells; in contrast, M2 coculture promotes A549 cell growth and invasion [7, 8].

E3 ubiquitin ligases are a large family of enzymes that play an essential role in catalysing ubiquitination by transferring ubiquitin from E2 ubiquitin-conjugating enzymes to substrates [9]. TRIM8 is a member of the TRIM family of proteins and is defined as an E3 ligase. TRIM8 is known to undergo K6-, K33-, K48-, and K63-linked ubiquitination, leading to proteasome-independent and proteasome-dependent responses [10–12]. Several reports have indicated the tumour suppressor role of TRIM8 through the activity of the p53 tumour suppressor protein and the occurrence of a feedback loop [13, 14]. However, some studies suggest the opposite view, suggesting that TRIM8, an oncogene, promotes NF-κB pathway activation [10]. However, the true role of TRIM8 in lung cancer has not been determined and requires further characterization.

Tumour metastasis is the process by which cancer cells disseminate from their origin to distant organs and is a complex process involving multiple stages, including cellular escape, intravasation into blood vessels, survival in the circulation, extravasation from blood vessels into secondary sites, and colonization [15]. In this study, we employed in vivo and in vitro experiments to determine the role and regulatory mechanisms of TRIM8 in lung cancer progression. Moreover, for the first time, we demonstrated that TRIM8 plays a pivotal role in suppressing lung cancer metastasis through ubiquitin-mediated degradation of MYOF. Our findings not only demonstrate the tumour suppressor role of TRIM8 but also provide evidence for its future clinical application in NSCLC treatment.

## Methods

### Study design and workflow

In previous work, we conducted microarray experiments to identify that TRIM8 expression was induced by M1 macrophages (antitumour macrophages). In this study, we further validated the cellular functions of TRIM8 through silencing with a TRIM8-specific shRNA and TRIM8 overexpression. These experiments were performed in A549 cells cocultured with M1 macrophages, and cell proliferation and migration were assessed. We expanded the scope by performing both in vitro and in vivo experiments to validate the suppressive functions of TRIM8. We also identified TRIM8-regulated genes by performing RNAseq experiments, followed by RT–qPCR validation and functional assays, including cell proliferation and migration assays. For the in vitro and in vivo experiments, we analysed at least three biological replicates per group to ensure reliable statistical power and capture the effect of variability in TRIM8 expression on cell proliferation, colony formation, migration and invasion. The results of RNAseq experiments were further validated by RT–qPCR experiments. With a medium effect size of 0.5, alpha error probability of 0.05, and power of 0.8, a minimum of 28 paired normal tissue–tumour samples were required to detect significantly lower expression in tumours. We also evaluated TRIM8 expression in clinical samples included public datasets (GSE19188, GSE30219, and GSE31210). Furthermore, we also examined the clinical outcomes associated with TRIM8 expression using both our Taiwanese lung adenocarcinoma cohort (*n* = 68) and publicly available datasets (Kaplan–Meier Plotter database). The detailed study workflow is shown in Supplementary Fig. S1.

### Cell culture and patient specimens

The human NSCLC cell lines A549 and H358 were obtained from the American Type Culture Collection (ATCC; Manassas, VA, USA), and CL1-0 was provided by Dr. Pan-Chyr Yang (Department of Internal Medicine, National Taiwan University Hospital, Taiwan). The cell culture procedures followed ATCC recommendations and previous reports [7, 16]. All patients underwent surgical resection of lung adenocarcinoma between September 2017 and March 2021 at the Chung Shan Medical University Hospital. The institutional review board of the hospital approved this study on 2022/02/24 (IRB CSMUH No: CS2-20206). Written informed consent was obtained from each participant in accordance with the Declaration of Helsinki.

The detailed materials and methods for the in vitro experiments, including cell transfection, proliferation assay, migration assay, invasion assay, co-immunoprecipitation, western blotting, and flow cytometry analysis, are provided in the supplementary methods section.

### Murine xenograft model

Specific pathogen-free (SPF) ASID mice were purchased from the National Laboratory Animal Centre (Taipei, Taiwan). The animal use protocols conformed to the guidelines of the Institutional Animal Care and Use Committee (IACUC) protocol and were approved by the ethics committee of National Chung Hsing University (Approval No: 109-111 ^R^). Mock control, oeTRIM8, shLacZ and shTRIM8 lung cancer cells (CL1-0 and H358: 2 × 10^6^ cells; A549: 1 × 10^6^ cells) were injected subcutaneously into the right posterior flanks of the mice to investigate the effects of TRIM8 on tumorigenesis and metastasis in vivo. After 6–8 weeks, the mice were sacrificed to observe the subcutaneous distribution of the tumours.

### Haematoxylin and eosin (H&E) staining

The tumour number was determined based on a gross visual inspection after serial sections were cut. For microscopic assessments, the mouse tissue samples were fixed with 10% neutral buffered formalin, processed in accordance with standard procedures, embedded in paraffin, and sectioned at a thickness of 4 μm. Staining followed standard H&E protocols using a Leica Autostainer XL combined with a CV5030 coverslipper (Leica Biosystems, Germany). The sections were deparaffinized in xylene and rehydrated through graded ethanol solutions (100% and 95%), followed by rinses with water. Nuclear staining was performed using Mayer’s haematoxylin, while the eosin staining was performed with a commercial eosin solution from Muto Pure Chemicals (Tokyo, Japan). Finally, the samples were dehydrated in graded alcohol solutions and cleared in xylene. The quantification of the area was based on measurements of digital slides [17].

### RNA sequencing analysis

RNA was quantified using SimpliNano™-Biochrom spectrophotometers (Biochrom, MA, USA). Sequencing libraries were generated using the KAPA mRNA HyperPrep Kit (KAPA Biosystems, Roche, Basel, Switzerland) according to the manufacturer’s recommendations. mRNA was purified from total RNA using magnetic oligo-dT beads. Synthesis of first-strand cDNA was performed using random hexamer primers. After generating double-stranded cDNA (dscDNA), to preferentially select cDNA fragments with a length of 300–400 bp, the library fragments were purified with a KAPA Pure Beads system (KAPA Biosystems, Roche, Basel, Switzerland). The library quality was assessed on a Qubit@ 2.0 Fluorometer (Thermo Scientific) and an Agilent Bioanalyzer 2100 system. Finally, the library was sequenced on the Illumina NovaSeq 6000 platform, and 150 bp paired-end reads were generated.

### Transcriptomics and pathway analysis

Initially, the programme Trimmomatic version 0.38 [18] was used to filter out the low-quality bases and adapter sequences in the raw data. Subsequently, the HISAT2 version 2.2.1 [19] was used to align the filtered reads with the GRCh38 reference genome, as previously described [20]. Moreover, the programme featureCounts version 2.0.0 [21] was used to quantify the transcript abundance and normalize the transcript per million (TPM) value. Finally, an R package called DEGseq (version 1.52.0) [22] was used to identify the differentially expressed genes (DEGs). In addition, Kyoto Encyclopaedia of Genes and Genomes (KEGG) pathway enrichment analyses of the differentially expressed genes were conducted using the DAVID Functional Annotation Tool Suite (https://david.ncifcrf.gov/) [23, 24]. The programme Genesis version 1.8.1 [25] was adopted for clustering and visualization of the gene clusters. A Venn diagram of the overlapping differentially expressed genes was produced using the VENNY 2.1 website (https://bioinfogp.cnb.csic.es/tools/venny/index.html) [26].

### Statistical analysis

The experimental values are presented as the means ± standard deviations (SDs). Before performing statistical analyses, the data distribution, within-group variation, and variance across comparison groups were analysed and evaluated. Appropriate statistical analysis methods were selected based on these evaluations. The significance of differences in functional assays between the control and TRIM8-transfected groups were evaluated using one-way analysis of variance (ANOVA) or a two-sample *t* test. For the clinical cohorts, a two-sample *t* test was applied when comparing *TRIM8* expression between normal and tumour samples, while a paired *t* test was used for *TRIM8* expression in paired normal tissues–tumour samples. Patients were stratified into two groups based on the median *TRIM8* expression (high- and low-expression groups) to examine the impact of *TRIM8* expression on the clinical outcomes of the 68 patients in our Taiwanese lung adenocarcinoma cohort. The Kaplan–Meier method was used to construct survival curves, and the log-rank test was applied to evaluate the differences between two groups. Additionally, univariate Cox proportional hazard regression analysis was performed to estimate the impact of *TRIM8* on clinical outcomes. We also analysed the association between *TRIM8* mRNA expression and clinical outcomes of patients with NSCLC and stage I disease using the Kaplan–Meier Plotter database (https://kmplot.com/analysis/index.php?p=service&cancer=lung) [27]. The statistical graphs were visualized with GraphPad Prism 9. All the statistical tests were two-tailed, and *P* < 0.05 was considered statistically significant.

## Results

### *TRIM8* was upregulated by M1 subtype macrophages in NSCLC

**Fig. 1:**
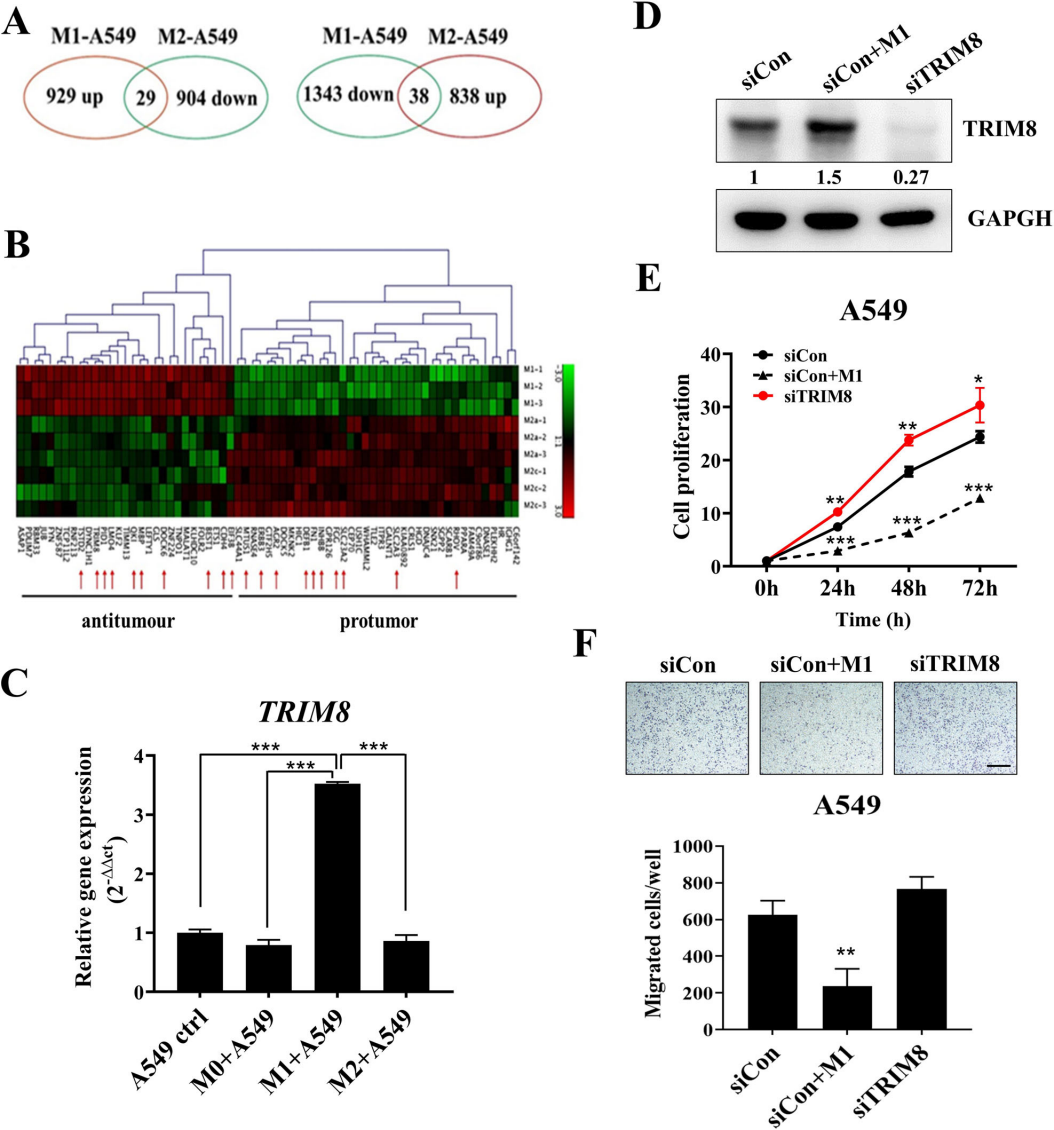
*TRIM8* is induced by M1 macrophages. **A** Venn diagram of the overlapping genes of A549 cells treated with polarized macrophage-conditioned medium (CM). A549 cells were cultured with M1 or M2 macrophage CM for 48 hours. Those genes that are upregulated by M1 macrophages and downregulated by M2 macrophages are classified as tumour suppressors (left panel). Those genes that are downregulated by M1 macrophages and upregulated by M2 macrophages are classified as oncogenes (right panel). **B** Heatmap of differentially expressed genes in macrophage-treated A549 cells. The red arrow indicates the top 20 genes. **C** Evaluation of *TRIM8* mRNA expression after culture in macrophage CM by RT‒qPCR. TATA-binding protein (*TBP*) was used as an internal control. **D** The expression of TRIM8 after M1 macrophage coculture and control or TRIM8 siRNA transfection, as detected by western blotting. The TRIM8 protein levels were quantified using ImageJ, with GAPDH serving as a control. **E** The viability of the cells in the silencing control (siCon), siCon with M1 macrophage coculture (siCon+M1), and TRIM8-silenced (siTRIM8) groups was evaluated via PrestoBlue™ reagent assays at the indicated times. **F** The cell migration of the siCon control, siCon with M1 macrophage coculture, and siTRIM8 cells, as determined by Transwell assay. Scale bar = 50 μm. The data are shown as the means ± SDs of at least three independent experiments, and the significance levels are indicated by *P < 0.05, **P < 0.01, and ***P < 0.001. The data were analysed with one-way ANOVA (**C**, **E**, **F**).

Based on the gene expression profiles of A549 cells treated with polarized macrophage-conditioned medium (CM) reported in our previous study [7], we screened differentially expressed genes regulated by M1 and M2 macrophages. Among the A549 control cells, genes that were significantly upregulated or downregulated by M1 or M2 macrophages in lung cancer cells and had more than twofold differential expression were filtered out. Among these genes, 929 M1-upregulated genes (defined by log_2_(M1-A549/A549) > 1.0) and 904 M2-downregulated genes (defined by log_2_(M2-A549/A549) < -1.0) were intersected to obtain 29 potential tumour suppressor genes (antitumour); in addition, 1343 M1-downregulated genes (defined by log_2_(M1-A549/A549) < -1.0) and 838 M2-upregulated genes (defined by log_2_(M2-A549/A549) > 1.0) were intersected to obtain 38 potential oncogenes (protumour) (Fig. 1A). Hierarchical clustering was performed for these two groups of genes, and the top 10 genes (red arrow) in each group were selected for further validation (Fig. 1B). The macrophage subtypes were characterized by RT‒qPCR (Supplementary Fig. S2). After the A549 cells were treated with M0 (unpolarized), M1 or M2 macrophage CM, the total RNA from the A549 cells was collected for differential gene expression analysis. We confirmed the expression of the above 20 genes by RT‒qPCR (data not shown). Among these genes, the expression of *TRIM8* mRNA was significantly induced by M1 macrophages (Fig. 1C), which provoked our interest. To test the effect of TRIM8 expression on cellular functions, we transiently silenced TRIM8 expression with TRIM8-specific siRNA or induced TRIM8 expression with M1 macrophage coculture in A549 cells. The western blot results indicated that TRIM8 expression was upregulated in A549 cells following coculture with M1 macrophages. Conversely, TRIM8 expression was reduced after silencing, regardless of whether a mixture of siRNAs or a single siRNA was used (Fig. 1D; Supplementary Fig. S3A). Compared to the siCon group, TRIM8 silencing significantly increased the proliferation of A549 cells and slightly increased their migration; conversely, M1 macrophage coculture increased the expression of TRIM8 and inhibited A549 cell proliferation and migration (Fig. 1E, F; Supplementary Fig. S3B and C). Given the antitumour effect of M1 macrophages, we hypothesized that TRIM8 might be a tumour suppressor.

### *TRIM8* expression is associated with the clinical outcomes of NSCLC patients

**Fig. 2:**
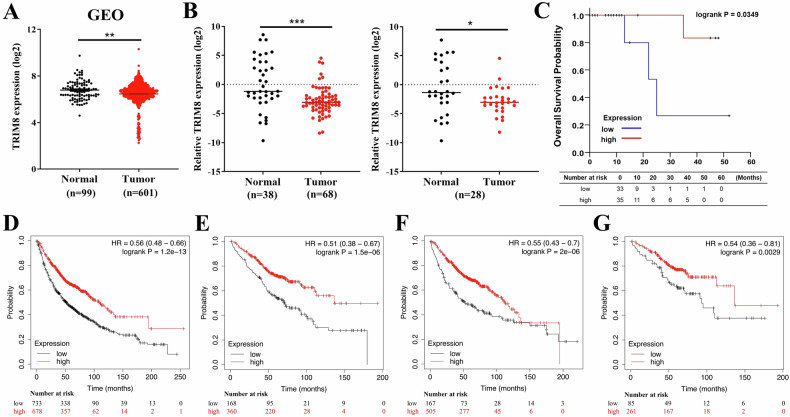
Correlation between TRIM8 expression and clinical outcome in patients with NSCLC. **A** *TRIM8* mRNA expression levels in 601 tumour tissues and 99 normal tissues from lung cancer patients in the GEO datasets (GSE19188, GSE30219 and GSE31210). The data were quantified by the log_2_ method. The data were analysed with *t* tests. **B** *TRIM8* mRNA expression in a Taiwanese lung cancer cohort comprising 68 lung cancer tissues and 38 adjacent normal tissues, as determined by real-time qPCR (left panel). The right panel shows *TRIM8* mRNA expression in 28 pairs of tumour tissue and normal tissue samples. The data were quantified by the log_2_ transformation of 2^−Δct^ values and are presented as the means ± SDs; *P < 0.05, *P < 0.01, and ***P < 0.001. The data were analysed with *t* tests (left panel) or paired *t t*ests (right panel). **C** Kaplan‒Meier curves for overall survival in 68 Taiwanese lung adenocarcinoma patients. Survival curves were also estimated and visualized by using published cohorts in the Kaplan‒Meier Plotter database. **D** Overall survival curve and univariate Cox regression analysis of 1411 NSCLC patients in the published cohort. **E** Overall survival curve and univariate Cox regression analysis of 528 patients with stage I NSCLC in the published cohort. **F** Overall survival curve and univariate Cox regression analysis of 672 lung adenocarcinoma patients in the published cohort. **G** Overall survival curve and univariate Cox regression analysis of 346 patients with stage I lung adenocarcinoma in the published cohort. The log-rank test was used to assess statistical significance, with a significance level set at *P* < 0.05.

We first analysed *TRIM8* mRNA expression in lung cancer patients using the Gene Expression Omnibus (GEO) database (GSE19188 [28], GSE30219 [29] and GSE31210 [30]) to explore the role of *TRIM8* in the clinical outcomes of lung cancer patients. GEO profiles of 601 lung cancer tissues and 99 adjacent normal tissues revealed lower levels of *TRIM8* mRNA in lung cancer tissues than in normal tissues (Fig. 2A). Next, we analysed 38 adjacent normal tissues and 68 lung cancer tissues from a Taiwanese cohort. The results revealed that the *TRIM8* mRNA level was markedly lower in lung cancer tissues than in normal tissues (Fig. 2B). In addition, the comparison of 28 pairs of tumour-normal tissue samples confirmed that the expression of *TRIM8* in tumour tissues was significantly reduced (Fig. 2B). The clinical characteristics of the sixty-eight Taiwan patients with lung adenocarcinoma are summarized in Supplementary Table S2. Subsequently, we evaluated the correlation between *TRIM8* expression and overall survival in the Taiwanese cohort and the published cohort from the Kaplan–Meier Plotter database. Analyses of 68 Taiwanese patients with lung adenocarcinoma showed that patients with low *TRIM8* expression experienced a significantly shorter overall survival time than did those with high *TRIM8* expression (*P* = 0.035; Fig. 2C). On the other hand, an analysis of published cohorts of NSCLC patients and patients with stage I NSCLC revealed that low expression of *TRIM8* was associated with shorter overall survival (*n* = 1411, *P* = 1.2E-13; Fig. 2D; *n* = 528, *P* = 1.5E–6; Fig. 2E). However, a significant association between low *TRIM8* mRNA expression and poor overall survival outcomes was predicted based on published data for cohorts of patients with lung adenocarcinoma (*n* = 672; *P* = 2E–6; Fig. 2F) and stage I disease (*n* = 346; *P* = 0.0029; Fig. 2G). Similarly, the published cohorts including patients with lung squamous cell carcinoma were predicted to have a poor overall survival outcome due to the low mRNA expression level of *TRIM8*, although the difference was statistically insignificant, as shown in Supplementary Fig. S4A (*n* = 527; *P* = 0.089). However, patients with stage I disease showed an opposite prognosis, and the difference was statistically insignificant (*n* = 137; *P* = 0.36; Fig. S4B). Moreover, all hazard ratios (HRs) of the univariate Cox regression analysis of the mRNA expression levels indicated the protective role of TRIM8 in the clinical outcomes of patients with NSCLC, except for patients with stage I lung squamous cell carcinoma. These results suggest that TRIM8 could play a tumour-suppressive role in affecting the clinical outcome of NSCLC.

### TRIM8 expression inhibited NSCLC cell proliferation and clonogenesis

**Fig. 3:**
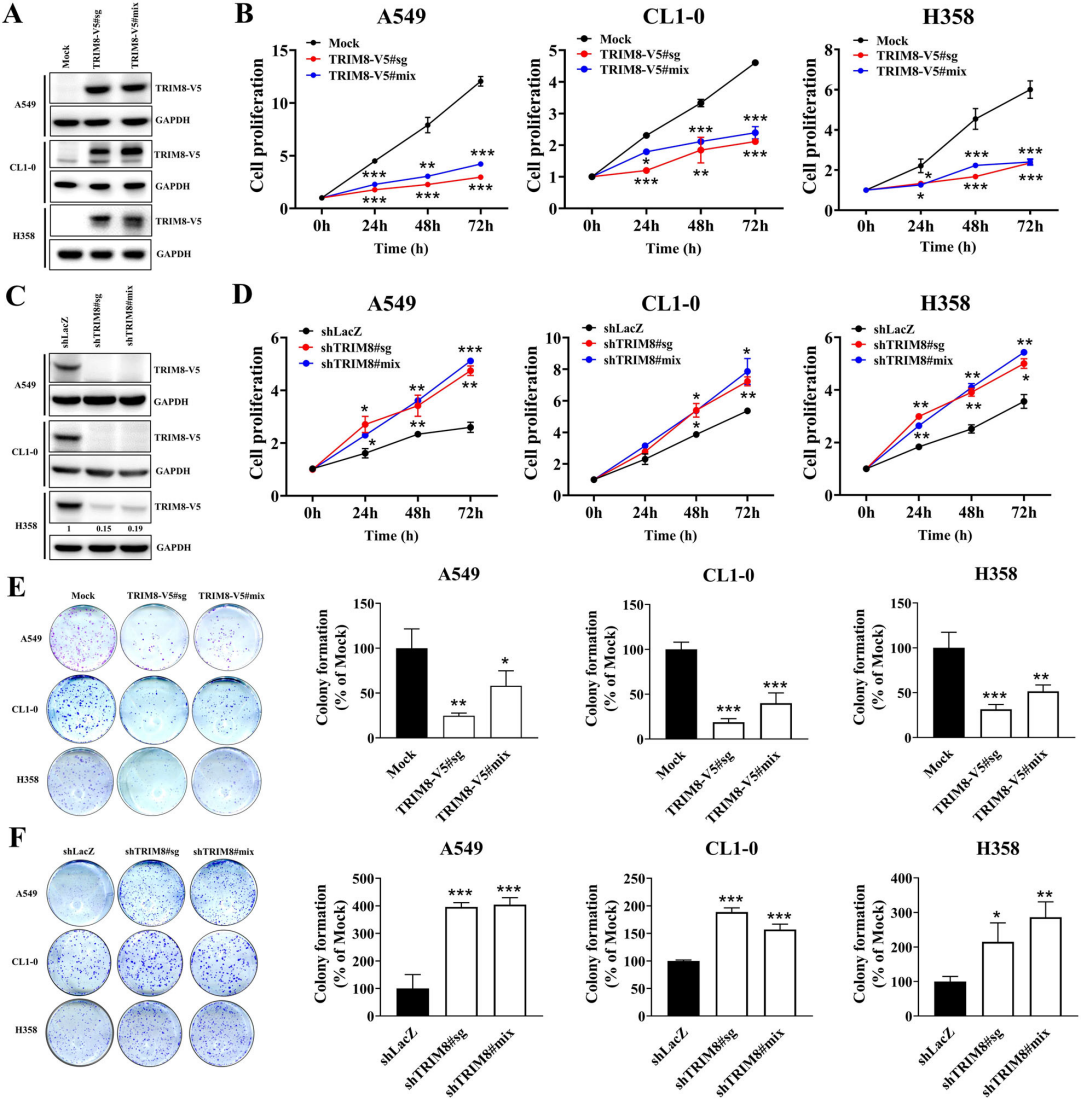
Effect of TRIM8 expression on lung cancer cell proliferation and colony formation. **A** Expression of the TRIM8-V5 protein in the transfectants, as measured through western blot analysis with an antibody against V5. The control cells (Mock) were transfected with the pcDNA3.1 vector. **B** The viability of mock, TRIM8-V5 single clone (#sg) and mixed clone (#mix) cells was evaluated by PrestoBlue™ reagent exclusion assays at the indicated times. **C** The silencing efficacy of the TRIM8-specific shRNA was determined by western blot analysis using an anti-V5 antibody. The exogenous TRIM8-V5 protein levels were quantified using ImageJ, with GAPDH serving as a control. **D** The viability of cells infected with the shLacZ or shTRIM8 virus was evaluated by PrestoBlue™ reagent exclusion assays at the indicated times. **E** Anchorage-dependent colony formation assay of *TRIM8* transfectants and mock transfectants. The colon-covered area in the experimental group was normalized to that in the mock control group. **F** Anchorage-dependent colony formation assay of shTRIM8 transfectants and shLacZ transfectants. The colony-covered area in the experimental group was normalized to that in the shLacZ control group. The data are presented as the mean ± SD of three independent experiments. *P < 0.05, **P < 0.01, and ***P < 0.001 compared with the mock or shLacZ control groups. The data were analysed with one-way ANOVA (**B**, **D**, **E**, **F**).

The lung cancer cell lines A549 (p53 wild-type), CL1-0 (p53^R248W^ mutant), and H358 (p53-null) were utilized in further studies to verify the function of TRIM8 in lung cancer cells. Cell clones with stable constitutive TRIM8 expression were established to elucidate the effects of TRIM8 expression on lung cancer progression. The results of the western blot analysis showed that all three stable clones of the cell lines expressed exogenous TRIM8-V5 (Fig. 3A). One mixed clone (oeTRIM8-V5#mix) and one single clone (oeTRIM8-V5#sg) that stably expressed TRIM8 were isolated for further investigations. As shown in Fig. 3B, increasing TRIM8 expression significantly inhibited the proliferation of A549, CL1-0, and H358 lung cancer cells. On the other hand, shRNA was used to silence TRIM8 protein expression in the TRIM8 transfectants and establish stable cell clones (Fig. 3C). One shLacZ control (shLacZ), one mixed clone (shTRIM8#mix), and one single clone (shTRIM8#sg) were isolated for subsequent studies. Instead, we found that, compared with the shLacZ control, shTRIM8 could effectively attenuate the TRIM8-mediated inhibition of cell proliferation (Fig. 3D). Furthermore, an in vitro anchorage-dependent assay was performed to evaluate the effect of TRIM8 on lung cancer cell growth. Compared with that in the mock control groups, the colony formation ability of the TRIM8-transfected cells was reduced by more than 50% in the A549, CL1-0, and H358 cell lines (Fig. 3E). In contrast, compared with shLacZ-transfected cells, shTRIM8-transfected cells exhibited a significant 1.5-fold to 4-fold increase in colony formation (Fig. 3F).

### TRIM8 restrained lung cancer cell migration and invasion

**Fig. 4:**
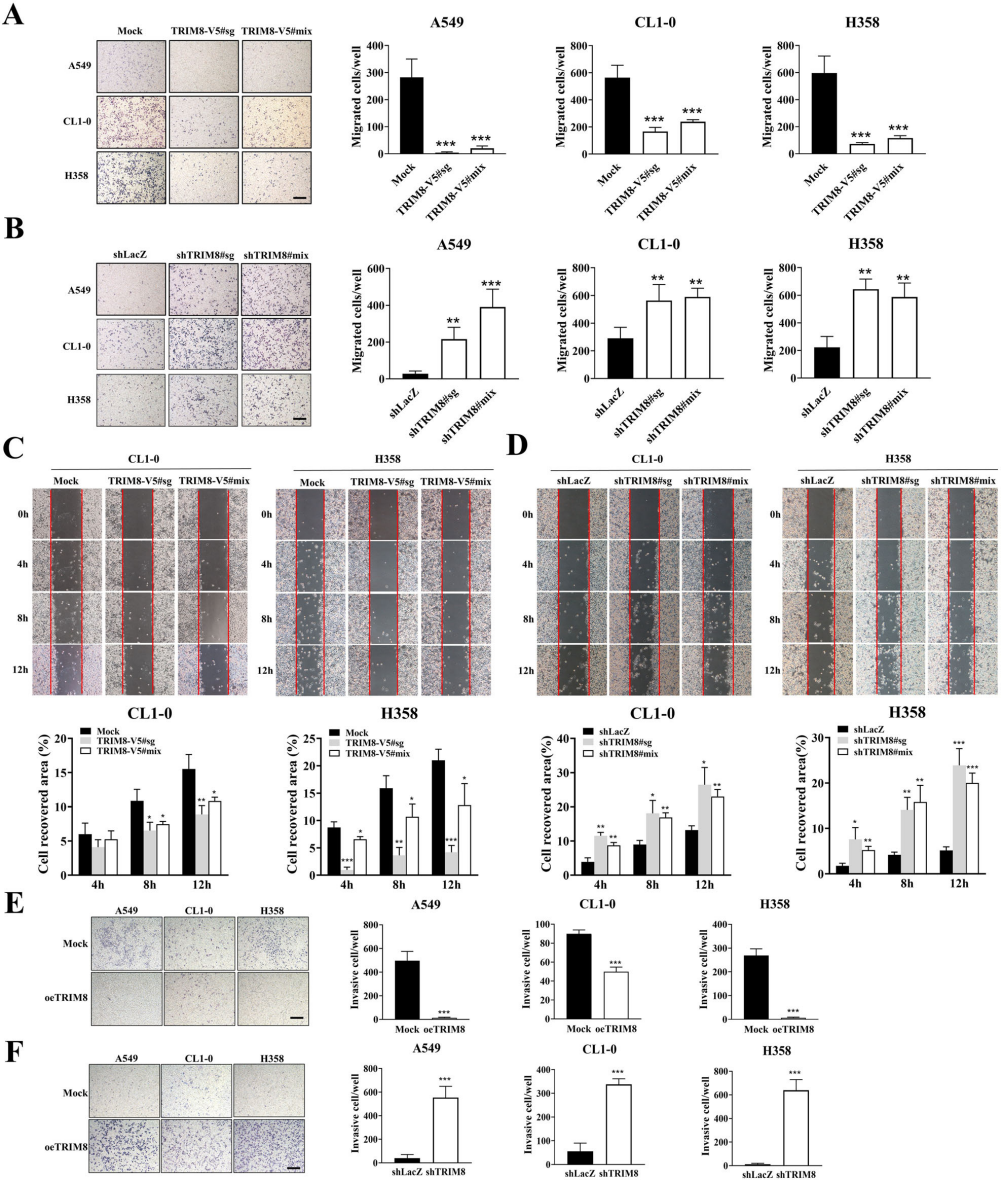
Effect of TRIM8 expression on lung cancer cell invasion and migration. **A** The vertical migration of mock and TRIM8-overexpressing transfectants was evaluated via Transwell assays. **B** The vertical migration of the shLacZ control transfectant and shTRIM8 transfectants was evaluated via Transwell assays. **C** The horizontal migration of the mock and TRIM8-overexpressing transfectants was assessed by a scratch wound assay, and wound closure was monitored at 0, 4, 8 and 12 h. Graphic quantification showed that TRIM8 overexpression significantly decreased wound closure. **D** The horizontal migration of the shLacZ control and shTRIM8 transfectants was assessed by a scratch wound assay, and wound closure was monitored at 0, 4, 8 and 12 h. **E** The invasion of the TRIM8 transfectants and mock transfectants was evaluated via Transwell assays. Scale bar = 50 μm. The data are shown as the means ± SDs of at least three independent experiments, and the level of significance was identified as *P < 0.05, **P < 0.01, and ***P < 0.001. The data were analysed with one-way ANOVA (**A**–**D**) or *t* tests (**E**, **F**).

To investigate the effect of TRIM8 on the vertical mobility of lung cancer cells, a Transwell assay was used. The vertical migration ability of the TRIM8 transfectants was markedly lower than that of the mock transfectants in the three different cell lines (Fig. 4A). Conversely, TRIM8 knockdown effectively reversed the inhibition of vertical cell motility resulting from TRIM8 compared with that of the shLacZ control (Fig. 4B). In addition, a scratch wound healing assay was utilized to investigate cell–cell interactions and horizontal cell migration. The results showed that, in CL1-0 and H358 cells, the horizontal mobility of the TRIM8 transfectants was significantly lower than that of the mock transfectants (Fig. 4C). Conversely, the TRIM8-mediated inhibition of horizontal cell migration was reversed in shTRIM8-transfected cells (Fig. 4D). For A549 cells, the wound healing effect was abolished because overexpression of TRIM8 disrupted the attachment ability of the cells (Supplementary Fig. S5B). Since invasion is the first step in the initiation of cancer metastasis, we assessed the effect of TRIM8 on cell invasion via a Matrigel invasion assay. We found that, compared with the mock control, the overexpression of TRIM8 in all three cell lines significantly inhibited cell invasion (Fig. 4E). This effect was reversed when TRIM8 expression was silenced. In all three cell lines, shTRIM8 transfection led to significant improvement in cell invasion (Fig. 4F).

### TRIM8 attenuated NSCLC tumorigenesis and metastasis in vivo

**Fig. 5:**
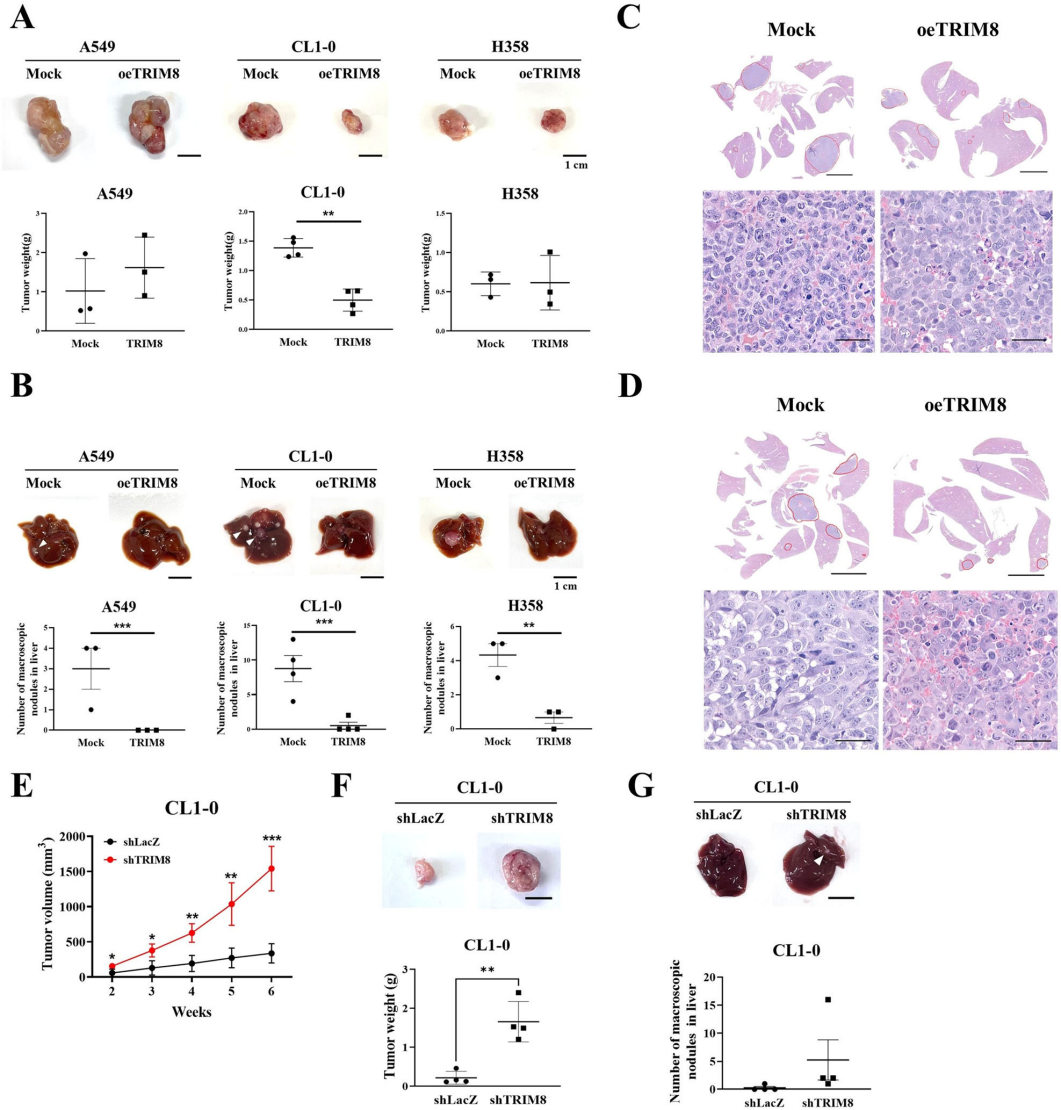
Overexpression of TRIM8 suppressed tumour metastasis in a xenograft model. **A** In vivo experiment in which A549, CL1-0 or H358 lung cancer cells were subcutaneously injected into a xenograft model (upper panels). The number of mice in each group was as follows: *n* = 3 for A549 and H358 cells and *n* = 4 for CL1-0 cells. Tumour weights of the mock control group and TRIM8-overexpressing group (oeTRIM8) after 49 days (A549 cells) or 56 days (CL1-0 and H358 cells) are shown in the lower panels. Scale bar = 1 cm. **B** Representative images of livers harvested from the mock control and oeTRIM8 animal groups (upper panels). Quantitative analysis of the total number of tumour nodules that metastasized from subcutaneous tumours to all liver regions was performed. Arrowheads indicate metastatic nodules. Scale bar = 1 cm. **C** Histopathological findings of the liver via subcutaneous injection of CL1-0 lung cancer cells in mice. Scale bar (upper panel) = 5 mm; scale bar (lower panel) = 50 μm. **D** Histopathological findings of the liver after subcutaneous injection of H358 lung cancer cells in mice. Circles show the tumour area. Scale bar (upper panel) = 5 mm; scale bar (lower panel) = 50 μm. H&E staining, ×0.4 and ×20. **E** Quantification of tumour volumes in solid tumour-bearing mice from the shLacZ and shTRIM8 groups over a 6-week period. The number of mice in each group was 4. **F** In vivo experiments were performed by subcutaneously injecting CL1-0 lung cancer cells into a xenograft model (upper panel). Quantification of tumour volumes in solid tumour-bearing mice from the shLacZ and shTRIM8 groups over a 6-week period (lower panel). Scale bars = 1 cm. **G** Representative images of livers harvested from animals in the shLacZ control and shTRIM8 groups (upper panel). A quantitative analysis of the total number of tumour nodules that metastasized from subcutaneous tumours to all liver regions was performed. Arrowheads indicate metastatic nodules (lower panel). Scale bars = 1 cm. The data are presented as the means ± SDs. **P < 0.01 and ***P < 0.001. The data were analysed with *t* tests.

In a subcutaneous xenograft model, we observed NSCLC tumorigenesis and metastatic progression by implanting TRIM8-overexpressing cells (oeTRIM8#mix clones) and control cells into ASID mice. Compared to the mock control group, the TRIM8-transfected group exhibited a 2.7-fold decrease in tumour weight (Fig. 5A), but subcutaneous tumour growth was not different between the A549 and H358 cells (Fig. 5A). However, compared to the mock transfection, TRIM8 transfection inhibited the metastasis of subcutaneous tumour cells from all three cell lines to the liver, and the number of tumour nodules that formed in the liver was significantly reduced (Fig. 5B). Conversely, compared to shLacZ, TRIM8 knockdown significantly promoted tumorigenesis and slightly facilitated the spread of CL1-0 tumour cells to the liver (Supplementary Fig. 5E-G). Furthermore, microscopy confirmed that the total tumour area in the CL1-0 (Fig. 5C) and H358 (Fig. 5D) lung cancer cell lines in the mock control group was much larger than that in the TRIM8 transfectant group. Microscopically, the tumour consisted of large pleomorphic epithelioid tumour cells and focal spindle-shaped hyperchromatic cells arranged in a solid growth pattern. The tumour cells exhibited vesicular nuclei and prominent nucleoli with a high nuclear/cytoplasmic ratio. Our results suggest that TRIM8 potently suppresses tumour formation and metastasis in vivo.

### Identification of TRIM8 downstream target genes through RNA sequencing analysis

**Fig. 6:**
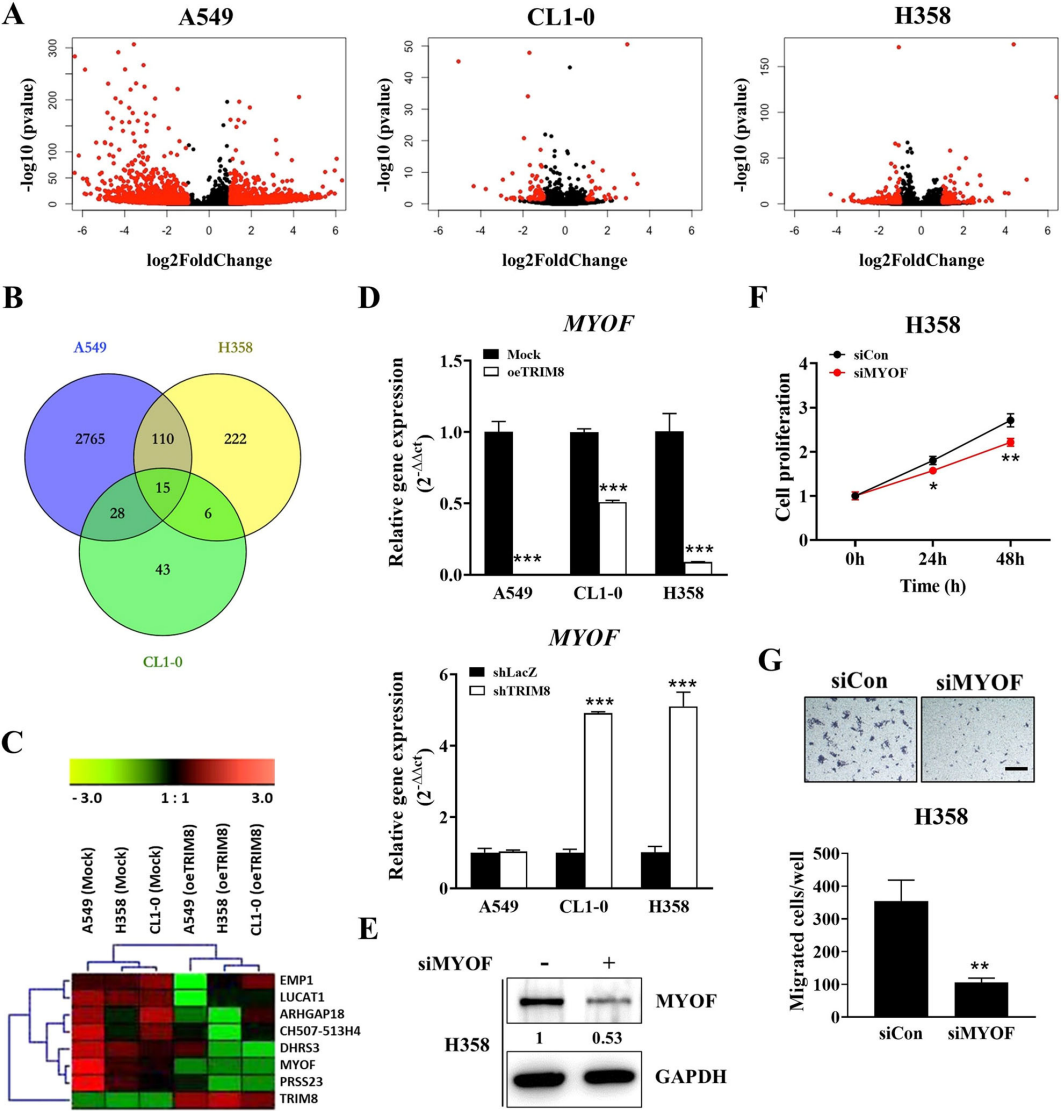
Downstream target genes regulated by TRIM8. **A** Volcano plot showing 2918, 92 and 353 differentially expressed genes in A549, CL1-0 and H358 cells (mock control vs. oeTRIM8#mix transfectant), respectively. **B** Venn diagram showing the overlapping differentially expressed genes in A549, CL1-0 and H358 cells. **C** A heatmap showing differential gene expression between mock and TRIM8 transfectants in three NSCLC cell lines. **D** *MYOF* mRNA expression in different transfectants was measured via RT‒qPCR. **E** Silencing of MYOF expression in H358 cells with a control siRNA or MYOF-specific siRNA. The protein level was detected by western blotting with the indicated antibody. The MYOF protein levels were quantified using ImageJ, with GAPDH serving as a control. **F** The viability of the silencing control (siCon) and MYOF-silenced H358 cells (siMYOF) was evaluated by PrestoBlue™ reagent exclusion assays at the indicated times. **G** Transwell migration assays were performed to evaluate the migration of siCon- and siMYOF-transfected H358 cells. Scale bar = 50 μm. The data are shown as the means ± SDs of at least three independent experiments. *TBP* was used as an internal control. The data were analysed with *t* tests; *P < 0.05, **P < 0.01, and ***P < 0.001.

To clarify how TRIM8 regulates NSCLC proliferation and migration, the genes differentially expressed between mock control and mixed TRIM8-overexpressing A549, CL1-0 and H358 cells were identified through RNA sequencing analysis. Compared with those in the control group, 2918, 92 and 353 transcripts whose expression differed by twofold were identified in A549, CL1-0 and H358 cells, respectively (*P* value < 0.05) (Fig. 6A). Furthermore, we overlaid the differentially expressed transcripts in the three cell lines and obtained 15 candidate transcripts, as shown in the Venn diagram (Fig. 6B), which included TRIM8. Among the 15 overlapping transcripts, 8 were consistently up- or downregulated by TRIM8 in all three cell lines compared with the respective mock controls (Fig. 6C). Among these genes, TRIM8 showed more than a twofold change in expression in the three TRIM8 transfectants (Supplementary Fig. S6A). The other seven transcripts (*EMP1*, *LUCAT1*, *ARHGAP18*, *CH507-513H4*, *DHRS3*, *MYOF*, and *PRSS23*) were downregulated in the three TRIM8 transfectants compared to the mock control-transfected cells, with more than twofold differences. Among these, *CH507-513H4* and *LUCAT1* were excluded because long noncoding RNAs (lncRNAs) do not encode proteins. We further verified the reliability of the results for the five transcripts in mock, TRIM8, shLacZ, and shTRIM8 transfectants (Fig. 6D, Supplementary Fig. S6). RT‒qPCR revealed that the overexpression of *TRIM8* decreased the *MYOF* (*Myoferlin*) mRNA level in the A549, CL1-0 and H358 cell lines. In contrast, TRIM8 knockdown reversed the changes in *MYOF* expression in CL1-0 and H358 cells but not in A549 cells (Fig. 6D). Previous studies have shown that *MYOF* is strongly associated with tumour metastasis and aggressiveness in breast cancer [31], clear cell renal cell carcinoma [32], and pancreatic ductal adenocarcinoma [33]. We used H358 cells with higher endogenous MYOF expression for the MYOF silencing experiments to confirm the functional importance of MYOF in NSCLC cell mobility (Fig. 6E). As anticipated, MYOF knockdown decreased the proliferation and migration of H358 lung cancer cells compared to the control group, regardless of whether a mixture or single siRNAs were used (Fig. 6F, G and Supplementary Fig. S7). These results were highly similar to those observed with TRIM8 overexpression. The KEGG analysis also revealed that the DEGs of the three transfected groups were significantly enriched in proteoglycans in cancer, focal adhesion, axon guidance, the MAPK signalling pathway, pathways in cancer, and other pathways (Supplementary Fig. S8). Therefore, because of our aforementioned inhibition of cell motility by TRIM8, we speculated that *MYOF* is a key downstream target gene of *TRIM8*.

### MYOF expression rescued the TRIM8-mediated inhibition of tumour migration

**Fig. 7:**
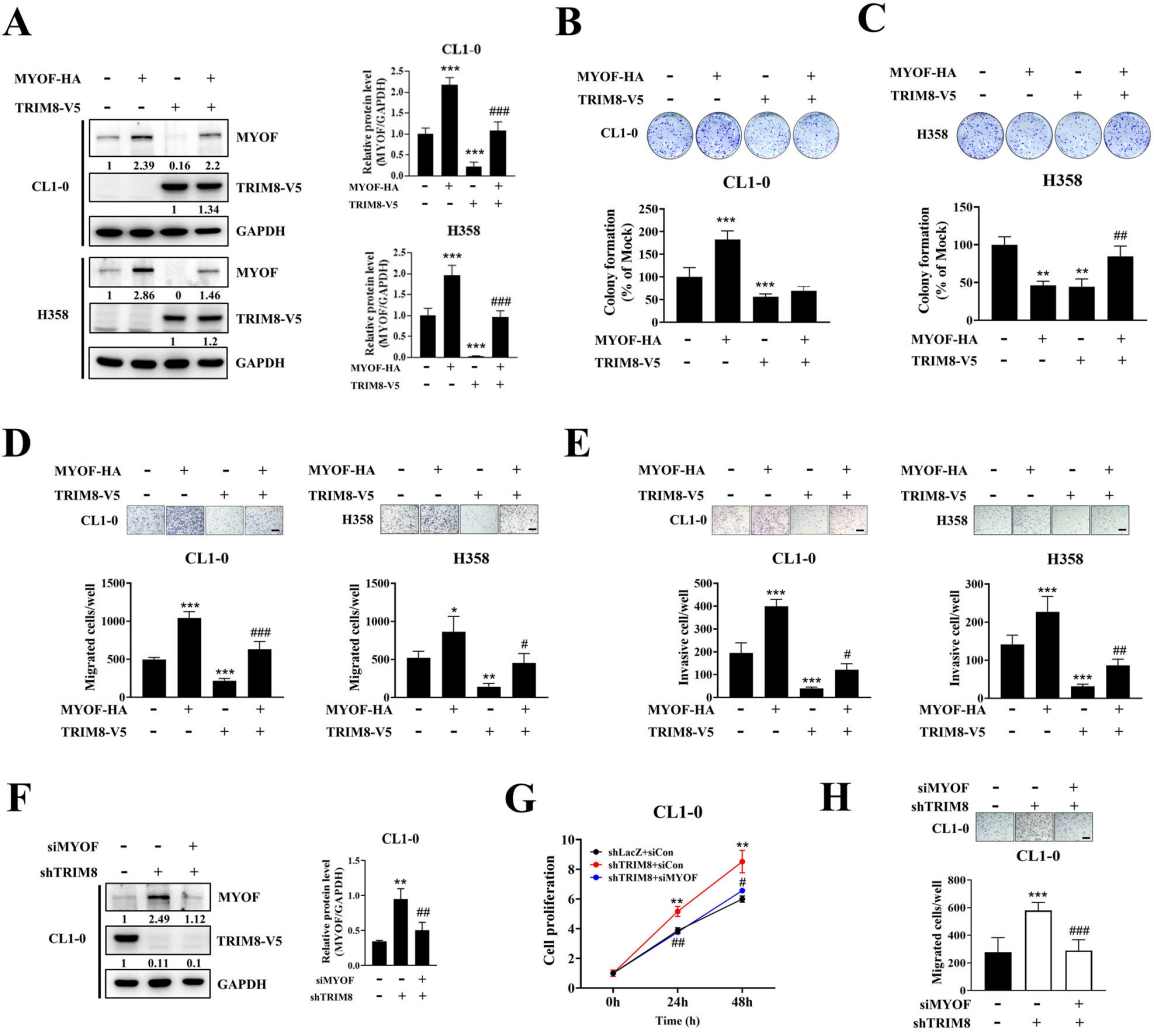
TRIM8 exerts its tumour-suppressive function by reducing MYOF expression. **A** CL1-0 and H358 cells stably expressing TRIM8-V5 were transfected with the MYOF-HA plasmid, and the expression levels of TRIM8-V5 and MYOF were detected by western blotting using the indicated antibodies. The protein expression levels were quantified using ImageJ, with GAPDH serving as a control. The relative levels of MYOF protein were normalized to GAPDH expression (right panel). **B**, **C** A colony formation assay was performed to detect the proliferation of CL1-0 and H358 cells. The colony-covered area was normalized to that in the mock control group. **D** A Transwell migration assay was performed to evaluate the migration of CL1-0 and H358 cells. **E** A Transwell invasion assay was performed to evaluate the invasion of CL1-0 and H358 cells. **F** Transfection of TRIM8-silenced CL1-0 transfectants with the control siRNA or MYOF siRNA. The expression levels of TRIM8-V5 and MYOF were detected via western blotting using the indicated antibodies. The protein expression levels were quantified using ImageJ, with GAPDH serving as a control. The relative levels of MYOF protein were normalized to GAPDH expression (right panel). **G** Viability of CL1-0 cells with or without double silencing of TRIM8 and MYOF, as determined using the PrestoBlue™ reagent. **H** Transwell migration assays were performed to detect the migration capability of CL1-0 cells with or without double silencing of TRIM8 and MYOF. Scale bar = 50 μm. The data are shown as the means ± SDs of at least three independent experiments. *P < 0.05, **P < 0.01, ***P < 0.001 compared with the mock or shLacZ control groups. ^#^*P* < 0.05, ^##^*P* < 0.01, and ^###^*P* < 0.001 compared with the TRIM8-overexpressing or TRIM8 knockdown groups. The data were analysed with one-way ANOVA.

To further determine whether MYOF is required for the TRIM8-mediated inhibition of lung cancer cell growth and migration, the pCDNA3.1-MYOF-HA plasmid was cotransfected into TRIM8-overexpressing CL1-0 and H358 cells. Western blot assays showed that MYOF was successfully overexpressed in mock- and TRIM8-transfected cells (Fig. 7A). In addition, the results confirmed that the MYOF protein level was dramatically suppressed by TRIM8, which was consistent with the RT‒qPCR results. Colony formation experiments showed that the effect of MYOF was inconsistent between CL1-0 and H358 cells. In CL1-0 cells, MYOF transfection significantly enhanced cell proliferation compared to that in the mock control group but did not rescue this inhibition in the context of TRIM8 overexpression (Fig. 7B). In contrast, MYOF transfection slowed cell proliferation compared to that in the mock control group of H358 cells but restored cell proliferation, which was inhibited by TRIM8 transfection (Fig. 7C). However, Transwell assays showed that MYOF overexpression increased the migration of both CL1-0 and H358 cells and rescued cell motility, which was inhibited by TRIM8 overexpression (Fig. 7D). Moreover, by performing Transwell assays with matrix gels, we confirmed that restoring MYOF expression rescued the invasive capability of lung cancer cells that was suppressed by TRIM8 (Fig. 7E). Furthermore, the western blot analysis of the shLacZ and shTRIM8 CL1-0 transfectants showed that the knockdown of TRIM8 expression could increase the MYOF protein level (Fig. 7F and Supplementary Fig. S9A). Silencing MYOF with a mixture of siRNAs or single siRNA in shTRIM8-transfected cells eliminated the TRIM8 deficiency-induced increases in cell proliferation and migration (Fig. 7G, H; Supplementary Fig. S9B and C).

According to previous reports, MYOF contributes to metastasis by increasing the expression of MMPs [34, 35]. Gelatine zymography was used to assess two key members of the matrix metalloproteinase family, MMP2 (gelatinase A) and MMP9 (gelatinase B). The western blot analysis revealed that TRIM8 overexpression did not change the cellular MMP2 or MMP9 protein levels (Supplementary Fig. S10). However, zymography of the MMPs showed that MMP2 and MMP9 activities were downregulated in the TRIM8-transfected cells (Supplementary Fig. S10). The above results suggest that TRIM8 suppresses the secretion of MMPs by depleting MYOF expression to inhibit cell migration.

### TRIM8 catalysed MYOF ubiquitination

**Fig. 8:**
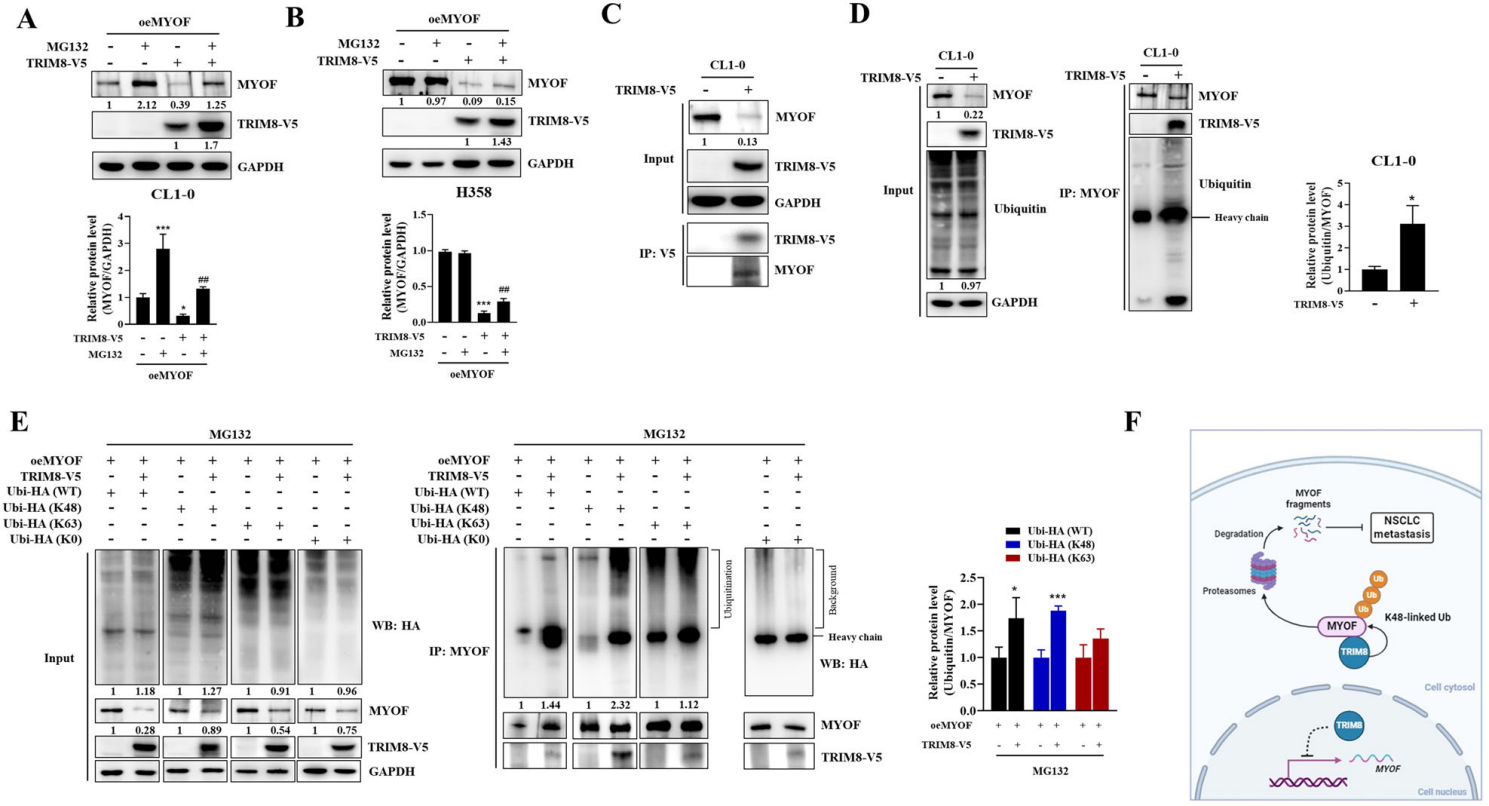
TRIM8 mediated MYOF degradation through the K48-ubiquitin‒proteasome pathway. **A**, **B** C1-0 and H358 mock and oeTRIM8 transfectants were transfected with the MYOF-HA plasmid and then treated with MG132 (10 μM) for 6 h before collection. The protein expression levels were quantified using ImageJ, with GAPDH serving as a control. The relative levels of MYOF protein were normalized to GAPDH expression (lower panel). **C** western blot analysis of the interactions between TRIM8 and MYOF in CL1-0 cells by coimmunoprecipitation with anti-V5 antibodies. The MYOF protein levels in the input groups were quantified using ImageJ, with GAPDH serving as a control. **D** Western blot analysis of the expression of endogenous MYOF and TRIM8-V5 (left panel). To measure the ubiquitination of endogenous MYOF, cell lysates were analysed via immunoprecipitation with ubiquitin antibodies followed by western blotting with anti-MYOF antibodies. The relative levels of ubiquitin protein were normalized to MYOF expression (right panel). **E** Cells were transfected with the MYOF-HA plasmid, wild-type (WT), or K48- or K63 or K0-ubiquitin plasmid and then analysed via immunoprecipitation with anti-MYOF antibodies followed by western blotting with antibodies against the HA tag to measure the polyubiquitination of MYOF. The cells were treated with MG132 (10 μM) for 6 h before collection. The ubiquitin-HA and MYOF protein levels in input were quantified using ImageJ, with GAPDH serving as a control (left panel). The relative levels of ubiquitin-HA protein in the IP group were quantified and normalized to MYOF expression (right panel). The above data were obtained from three independent experiments. **F** A schematic model of TRIM8-mediated inhibition of NSCLC metastasis. The dashed line represents an unclear mechanism.

Since TRIM8 is an E3 ligase, to advance the understanding of the regulatory mechanism between TRIM8 and MYOF, we first investigated whether MYOF is degraded by proteasome-dependent proteins. The CL1-0 and H358 transfectants were transfected with the MYOF plasmid before being treated with or without MG132. The results showed that MG132 significantly increased the MYOF protein level, which was suppressed by TRIM8 in CL1-0 and H358 cells, even when TRIM8 was upregulated (Fig. 8A, B). Interestingly, MG132 treatment did not rescue the decrease in endogenous MYOF levels induced by TRIM8 without transfection with the MYOF plasmid (Supplementary Fig. S11). We performed a coimmunoprecipitation assay to confirm the interaction between the TRIM8 and MYOF proteins. The results showed that exogenous TRIM8-V5 could bind to endogenous MYOF (Fig. 8C). Moreover, exogenous TRIM8-V5 was also coimmunoprecipitated by endogenous MYOF in CL1-0 cells; compared with that in the mock control cells, the amount of ubiquitin labelled on MYOF was higher in the TRIM8-transfected cells (Fig. 8D). We transfected cells with wild-type ubiquitin (WT), ubiquitin lysine-free (K0), ubiquitin only lysine 48 (K48) or lysine 63 (K63), or other lysine residues mutated to arginine to identify the linkage types of the ubiquitin chains of MYOF mediated by TRIM8. Compared to those in the respective mock controls, the overall levels of wild-type ubiquitin and K48-type ubiquitin were increased in the TRIM8 transfectants (Fig. 8E, left panel). Wild-type, K48-type, K63-type and K0-type ubiquitin were all present in the coimmunoprecipitates of MYOF from both the TRIM8 transfectant and mock control; compared to the respective mock controls, the wild-type and K48-type ubiquitination was more pronounced in the MYOF coimmunoprecipitate of the TRIM8 transfectants (Fig. 8E, right panel). This result indicated that during the process by which TRIM8 ubiquitinates MYOF, K48-linked ubiquitination is the predominant mechanism.

## Discussion

Several studies have substantiated the involvement of the E3 ubiquitin ligase TRIM8 in various diseases and tumorigenesis [14, 36–38]. However, the underlying mechanisms and the specific substrates regulated by TRIM8 remain to be fully elucidated. This study is the first to identify the novel TRIM8-interacting protein MYOF and to clarify that TRIM8 regulates lung cancer metastasis through the K48-linked ubiquitin-mediated degradation of MYOF.

As a member of the TRIM family of proteins, TRIM8 has various functions in cellular processes, including intracellular signalling, apoptosis [39], the immune response [36, 37] and carcinogenesis [14, 40]. Interestingly, in this study, we found that the expression of TRIM8 is induced by M1 macrophages. We believe that the relationship between TRIM8 and the tumour microenvironment in lung cancer is worthy of further exploration. Previous studies revealed its dual role in human cancer. As a tumour suppressor, TRIM8 stabilizes the p53 protein, leading to cell cycle arrest and a reduction in cell proliferation [13]. A recent report indicated that TRIM8 suppressed tumorigenesis by repressing the Wnt5a/β-catenin signalling pathway and induced apoptosis by activating the caspase-3 signalling pathway [38]. However, our results indicate that the expression levels of TRIM8 in the CL1-0 stable clones have no effect on the cell cycle or apoptosis (Supplementary Fig. S13A–D). Nevertheless, after treating the CL1-0 stable clones with hydrogen peroxide (H_2_O_2_), TRIM8 depletion attenuated H_2_O_2_-triggered apoptosis (Supplementary Fig. S13E and F). This result is consistent with previous studies showing that TRIM8 knockdown protects HK-2 (human renal proximal tubular epithelial cells) cells by alleviating oxidative stress-mediated apoptosis [39]. Although TRIM8 has different functions in various cancer types, its function in lung cancer is still unclear and requires further investigation. Our findings may lead to the use of a new prognostic factor, TRIM8, in NSCLC patients and provide a new reference for clinical treatment.

Our in vitro experiment revealed that TRIM8 could inhibit lung cancer cell growth and motility, which was reversed by TRIM8 silencing. Previous studies have shown that TRIM8 reduces cell proliferation by stabilizing the p53 protein [13]. However, the cell lines used in this study (A549, CL1-0 and H358) have different p53 genotypes, and our results suggest that the regulation of tumour progression by TRIM8 may not be mediated by p53. Moreover, we observed that, compared with the shLacZ control, TRIM8 shifted the morphology of A549 lung cancer cells from fibroblast-like to round and dense, while TRIM8 knockdown decreased the A549 cell density and increased cell extensibility (Supplementary Fig. S5A). In addition, we discovered that TRIM8 strongly attenuated the attachment ability of A549 cells, whereas silencing TRIM8 reversed this effect (Supplementary Fig. S5B). This is why we excluded A549 cells from the assay; thus, the underlying mechanism needs to be further investigated in the future to clarify the relationship between TRIM8 and cell adhesion. Furthermore, in a murine xenograft model, TRIM8-overexpressing cells decreased the formation of tumour nodules that metastasized from subcutaneous tumours to the liver in all the A549, CL1-0 and H358 transfectants (Fig. 5B). However, only the overexpression of TRIM8 in CL1-0 cells suppressed tumorigenesis in situ (Fig. 5A). Notably, although three cell lines were used to overexpress the TRIM8 protein, there were slight differences in cell characteristics and in vivo experiments.

In this study, we first identified TRIM8 as a tumour suppressor in human lung adenocarcinoma. Our results showed that, compared with high expression, low *TRIM8* mRNA expression is associated with shorter overall survival in patients with NSCLC, early-stage lung adenocarcinoma and squamous cell carcinoma. Therefore, our results suggest that TRIM8 could play a protective role in terms of the clinical outcomes of NSCLC and early-stage disease. To further investigate the mechanism underlying the TRIM8-mediated suppression of tumour progression, RNA-seq was performed to identify a promising candidate gene, MYOF, that is involved in the regulatory effects of TRIM8. MYOF is a protein of the ferlin family that is highly expressed in skeletal muscle, heart muscle, and endothelial cells. Recently, MYOF was identified as a promising biomarker for various cancers, including NSCLC [41]. MYOF has been reported to be involved in proliferation [32], metastasis [32], angiogenesis [42], and the epithelial–mesenchymal transition [43] in various cancer cells. TRIM8 might regulate cell growth and movement by several different mechanisms. We selected the *MYOF* gene as our main point of discussion regarding the relationship between TRIM8 and cell motility. Our data showed that TRIM8 overexpression could significantly reduce *MYOF* expression and vice versa, suggesting that MYOF may be a downstream target of TRIM8. This suggestion was also supported by the migration and invasion assays, which showed that the MYOF-induced increase in migration and invasion could be reversed by TRIM8 overexpression (Fig. 7C and J). Conversely, MYOF depletion suppressed cell mobility (Fig. 7F), even in TRIM8-knockdown cells (Fig. 7I). However, the impact of MYOF on colony formation was inconsistent between CL1-0 and H358 cells, suggesting that TRIM8 may not regulate cell proliferation through MYOF. These results confirmed that TRIM8 impairs cell motility and invasion by inhibiting the expression of MYOF; on the other hand, it may affect cell proliferation through an independent pathway, which needs further study.

Previous studies have shown that MYOF depletion can suppress metastasis and reverse the epithelial-to-mesenchymal transition [34, 44]. The decrease in cell invasion mediated by MYOF knockdown may partially result from the downregulation of several matrix metalloproteinases (MMPs), such as MMP2 [34]. MMPs are a family of proteases that degrade components of the extracellular matrix and are well known for their association with cancer metastasis. Our data revealed that TRIM8 expression inhibits MYOF expression and further reduces the secretion of MMP2 and MMP9, resulting in decreased cell migration and invasion, which is also in line with the findings of previous reports. In this study, we first revealed that TRIM8 inhibits cell migration by regulating MYOF, which is regulated at both the RNA and protein levels. TRIM8 enhances K48-linked ubiquitination of the MYOF protein and mediates MYOF protein degradation via the ubiquitin‒proteasome pathway. Previous studies have shown that TRIM8 promotes the K63-linked polyubiquitination of TAK1, leading to the activation of TAK1 and enhanced inflammatory responses [10]. In our study, overexpression of TRIM8 resulted in a slight increase in K63-linked ubiquitination of MYOF, but this change occurred predominantly through K48-linked ubiquitination. Recent research has demonstrated that TRIM8 catalyses the translation of TRIM21 via K48-linked polyubiquitination [45]. This is the first report to indicate that TRIM8 degrades its substrate, MYOF, through K48-linked ubiquitination to inhibit lung cancer cell mobility. Treatment with MG132 fully or partially restored the suppressive effect of exogenous MYOF on TRIM8, but it failed to rescue the levels of endogenous MYOF (Fig. 8A, B). We speculate that this difference is due to the strong suppression of *MYOF* mRNA expression by TRIM8, but how TRIM8 mediates *MYOF* mRNA expression is still unknown. TRIM8 contains a nuclear localization signal at the C-terminus, which allows it to translocate into the nucleus. Our results also showed that TRIM8 is expressed in both the nucleus and the cytoplasm (Supplementary Fig. S12). A previous report revealed that TRIM8 is predominantly expressed in the nucleus and stabilizes phosphorylated IRF7. Knockdown of TRIM8 diminishes IRF7-induced IFN production in plasmacytoid dendritic cells [46]. This finding indicates that TRIM8 may play a critical role in the nucleus, but the underlying mechanism is unclear. In addition, previous studies have indicated that TRIM8 regulates the transcription factors p53 [13], NF-κB [10, 37], and STAT3 [47], playing dual roles in tumour progression. Our results showed that TRIM8 potently represses MYOF mRNA and protein expression (Figs. 6D, 7A). Therefore, the speculation that TRIM8 may directly or indirectly modulate *MYOF* mRNA expression through transcriptional regulation is reasonable. In further investigations, we will explore how TRIM8 regulates *MYOF* mRNA expression in the nucleus.

While this study provides novel insights into the mechanism by which TRIM8 regulates MYOF to affect the progression of non-small cell lung cancer, the precise role of TRIM8 as a tumour suppressor within the nucleus and its regulation of the *MYOF* mRNA remain to be elucidated. Additionally, the regulatory interactions between TRIM8 and the tumour microenvironment during lung cancer progression are not yet clearly defined, such as how M1 macrophages induce TRIM8 expression. Moreover, further validation using larger clinical cohorts is required to establish the reliability of TRIM8 as a robust prognostic biomarker in clinical settings.

In summary, this study revealed that TRIM8 suppresses lung cancer cell proliferation, colony formation, migration, invasion, and metastasis. The expression of TRIM8 in lung cancer tissues was lower than that in normal tissues and was negatively correlated with patient survival. Our findings definitively indicate that TRIM8 constrains the progression of non-small cell lung cancer by facilitating the degradation of MYOF in the cytoplasm via K48-linked ubiquitination. However, the mechanism by which TRIM8 inhibits MYOF transcription in the nucleus still requires further exploration (Fig. 8F). Overexpression of MYOF reversed the inhibitory effect of TRIM8 on the motility of lung cancer cells in vitro. Moreover, MMP expression was reduced due to MYOF depletion. Taken together, our work indicates that TRIM8 inhibits lung cancer cell motility, at least in part, through the TRIM8-MYOF-MMP axis. These findings suggest that restoring TRIM8 expression could serve as a novel therapeutic strategy and biomarker for lung cancer. For instance, screening for compounds that upregulate TRIM8 expression or developing proteolysis targeting chimaeras (PROTACs) to enhance TRIM8 targeting of the MYOF protein could provide potential therapeutic options.

## Supplementary information

Supplementary Information

Raw data of western blot

## Data Availability

The accession number of the RNA-seq data is GSE229282. All the data can be found in the main text or in the supplementary material.
