# Supplementary material for: E3 ligase TRIM8 suppresses lung cancer metastasis by targeting MYOF degradation through K48-linked polyubiquitination
Source: Cell Death Dis. 2025 Feb 11;16(1):88. doi: 10.1038/s41419-025-07421-6 (PMC11814372; doi:10.1038/s41419-025-07421-6)
Supplement: Supplementary file 1 — Supplementary Information [file 41419_2025_7421_MOESM1_ESM.docx]

**Supplementary** **Information**

**E3 ligase TRIM8 suppresses lung cancer metastasis by targeting MYOF degradation through K48-linked polyubiquitination**

Chi-Hsuan Wei, Chia-Wei Weng, Chih-Ying Wu, Hsuan-Yu Chen, Ya-Hsuan Chang, Gee-Chen Chang, and Jeremy J.W. Chen

Correspondence: Professor JJW Chen, E-mail: jwchen@dragon.nchu.edu.tw

**Supplementary Table S1. DNA primers used to quantify gene expression**

| **Target** | **Strand** | **Sequence (5’→3’)** |
| --- | --- | --- |
| **TBP** | Forwards | ACGAACCACGGCACTGATTT |
|  | Reverse | GGAAAACCCAACTTCTGTACAACTC |
| **GAPDH** | Forwards | CATGGGTGTGAACCATGAGAAGT |
|  | Reverse | GGCATGGACTGTGGTCATGAG |
| **TRIM8** | Forwards | CGGAAGATGCTCATGAAGCA |
|  | Reverse | CCTCCTTCAGTTGGTTCACTTTCT |
| **IL-6** | Forwards | AGCCACTCACCTCTTCAGAACGA |
|  | Reverse | GTGCCTCTTTGCTGCTTTCAC |
| **IL-1β** | Forwards | CAGTGGCAATGAGGATGACTTG |
|  | Reverse | AGTGGTGGTCGGAGATTCGT |
| **IL-12β** | Forwards | ATTCGCTCCTGCTGCTTCACA |
|  | Reverse | CGTCCAGAATAATTCTTGGCCTC |
| **IL-10** | Forwards | ACCTGCCTAACATGCTTCGAGAT |
|  | Reverse | CCTTAAAGTCCTCCAGCAAGGA |
| **CD206** | Forwards | TACCCCTGCTCCTGGTTTTTG |
|  | Reverse | CACTGGGACTCACTGCATCCA |
| **ARHGAP18** | Forwards | GGAGCTGCCATTAGAATCAAGAA |
|  | Reverse | GGCAACTCCCGAATGAAGAG |
| **EMP1** | Forwards | GTGCGGTCACATACTTCCAGAA |
|  | Reverse | CAGCCAGCAATACCAACATGTT |
| **PRSS23** | Forwards | TCTTCTTTCTGCTCTGTGCTGTTG |
|  | Reverse | GCTTGGCTAAATTGAGGGTAGACT |
| **MYOF** | Forwards | GATGACCTGCTGGTTGTTGAGA |
|  | Reverse | CCCAATGCTGACTTCAAACTGA |
| **DHRS3** | Forwards | GGGCATGAGAGTCAGGTTTCC |
|  | Reverse | AACGAGGGCATGCATTGTC |
|  |  |  |

**Supplementary Table S2. Clinical characteristics of Taiwanese patients with lung adenocarcinoma**

| **Clinical characteristics** | **Taiwanese cohort** |
| --- | --- |
| No. of patients | 68 |
| Age, yrs | 64.25±9.37 |
| Gender – no. of patients (%) |  |
| Male | 28 (41.18) |
| Female | 40 (58.82) |
| Tumour stage, no. of patients (%) |  |
| I | 40 (58.82) |
| II | 12 (17.65) |
| III | 16 (23.53) |

**Supplementary Figure S1**


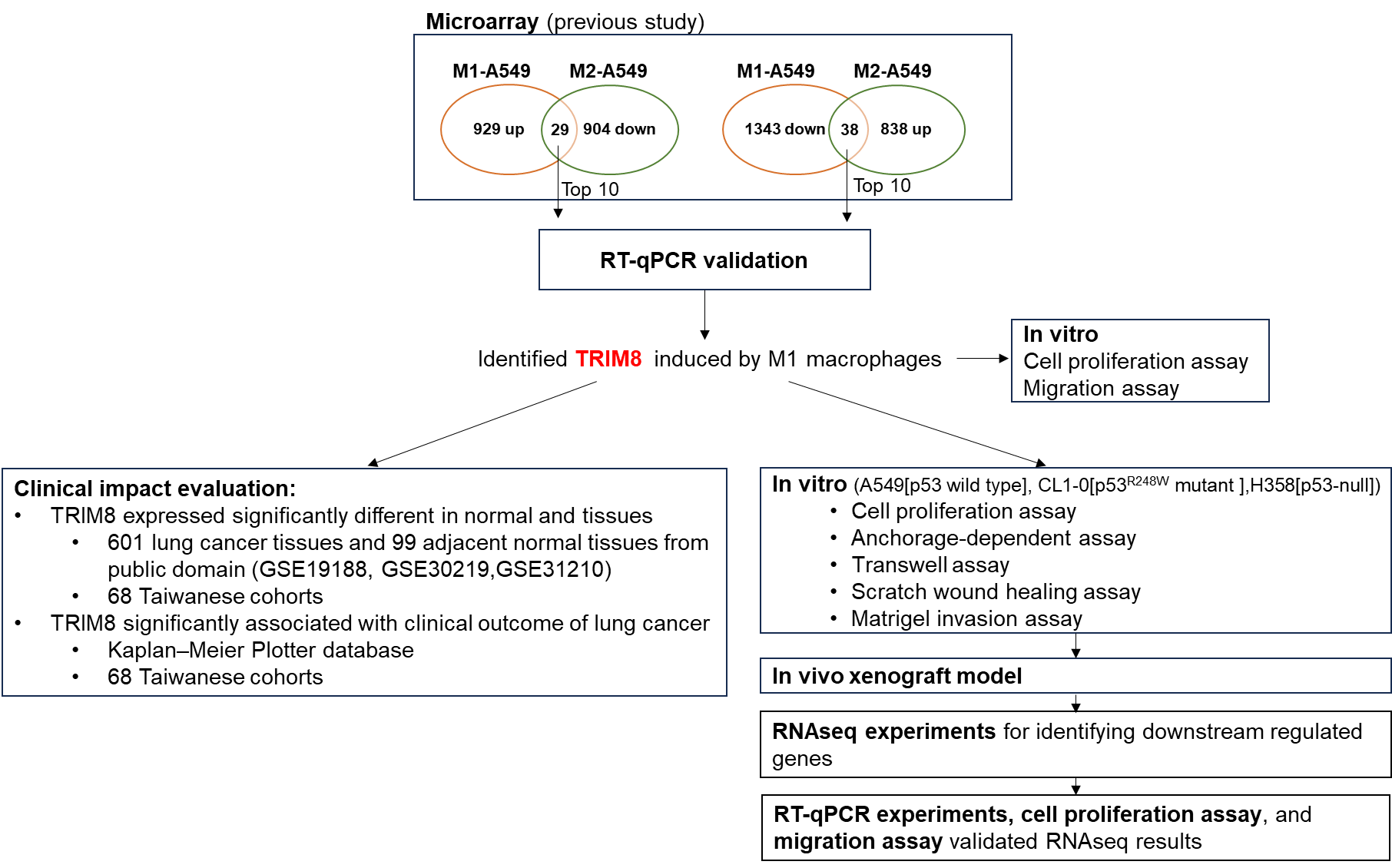


**Figure S1. The workflow of the whole study.**

**Supplementary Figure S2**

**
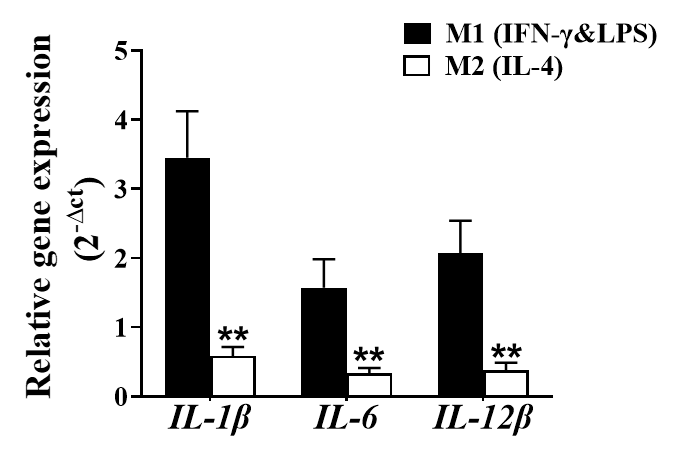

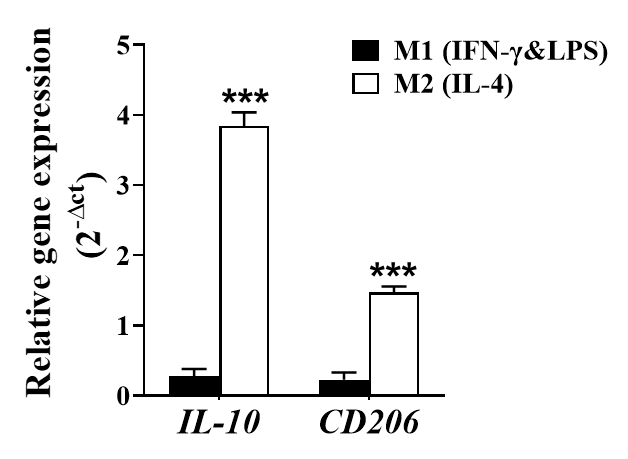
A B**

**Figure S2. Differential expression of Th1 and Th2 cytokines and M2 markers in differentiated macrophage subtypes.**

**A** The levels of Th1 cytokines in differentiated macrophage subtypes were determined by RT‒qPCR. **B** The expression of Th2 cytokines and M2 markers in differentiated macrophage subtypes was determined by RT‒qPCR. Gene expression in differentiated macrophage subtypes was normalized to that of untreated M0 macrophages. TATA-binding protein (*TBP*) was used as an internal control. The abovementioned data are presented as the means ± SDs of three independent experiments. ***P* < 0.01 and ****P* < 0.001.

**Supplementary Figure S3**

**
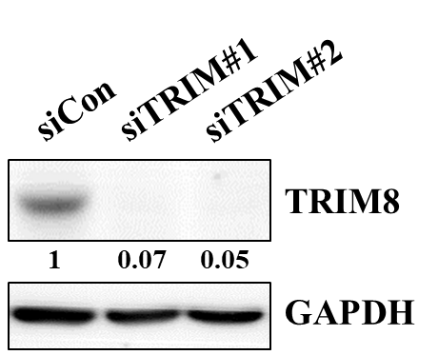

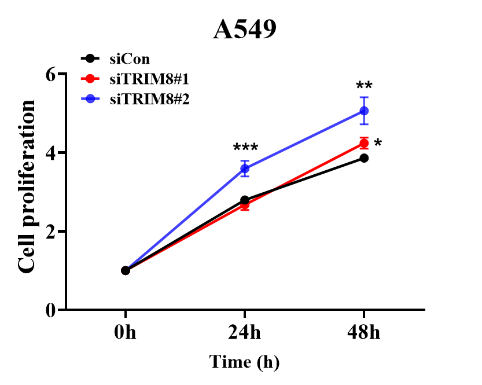
A B**

**
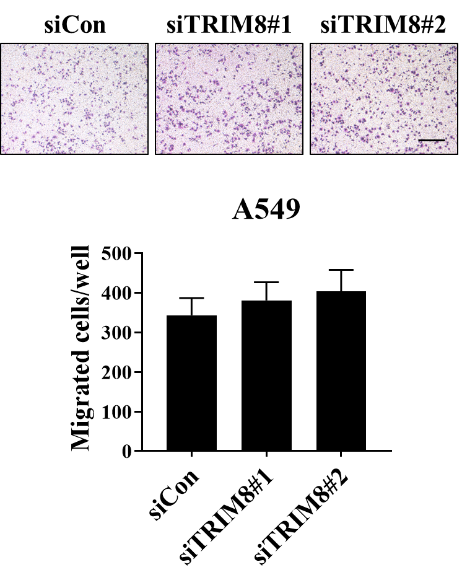
C**

**Figure S3. Silencing of TRIM8 expression in A549 cells using TRIM8-specific siRNAs.**

**A** The expression of TRIM8 in control siRNA-transfected (siCon) and two TRIM8-specific siRNA-transfected cells was detected by western blotting. The TRIM8 protein levels were quantified using ImageJ, with GAPDH serving as a control. **B** Viability of A549 cells following TRIM8 silencing, as determined with the PrestoBlue™ reagent. **C** Transwell migration assays were performed to assess the migratory ability of A549 cells following TRIM8 silencing. Scale bar = 50 μm. The abovementioned data are presented as the means ± SDs of three independent experiments. **P* < 0.05, ***P* < 0.01, and ****P* < 0.001 compared with the siCon control groups.

**Supplementary Figure S4**

**
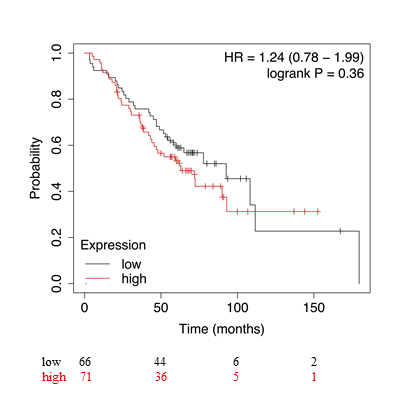

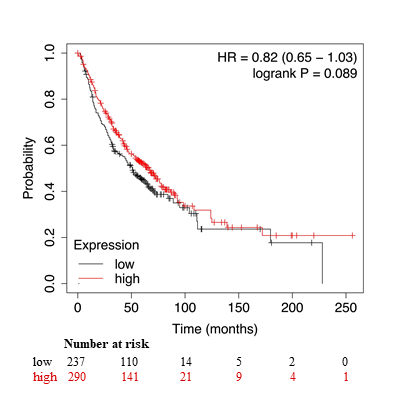
A B**

**Figure S4. Kaplan–Meier estimates and univariate Cox regression analyses of the overall survival of patients with lung squamous cell carcinoma stratified according to the TRIM8 expression level.**

Overall survival curves and univariate Cox regression analyses are shown for **A** 527 patients with lung squamous cell carcinoma and for **B** 137 patients in this cohort who had stage I disease. Survival curves were generated and visualized using the Kaplan‒Meier Plotter database. The statistical significance was evaluated using the log-rank test. *P* values less than 0.05 were considered to indicate statistical significance.

**Supplementary Figure S5**

**
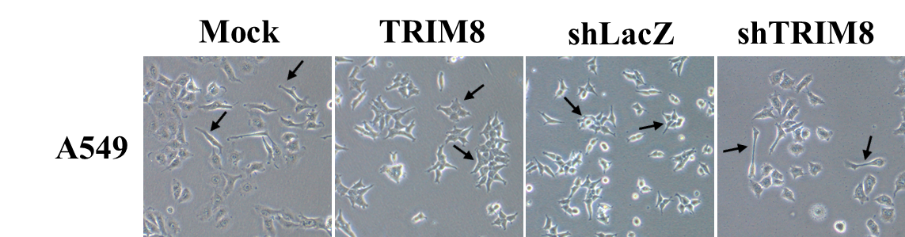
A**

**
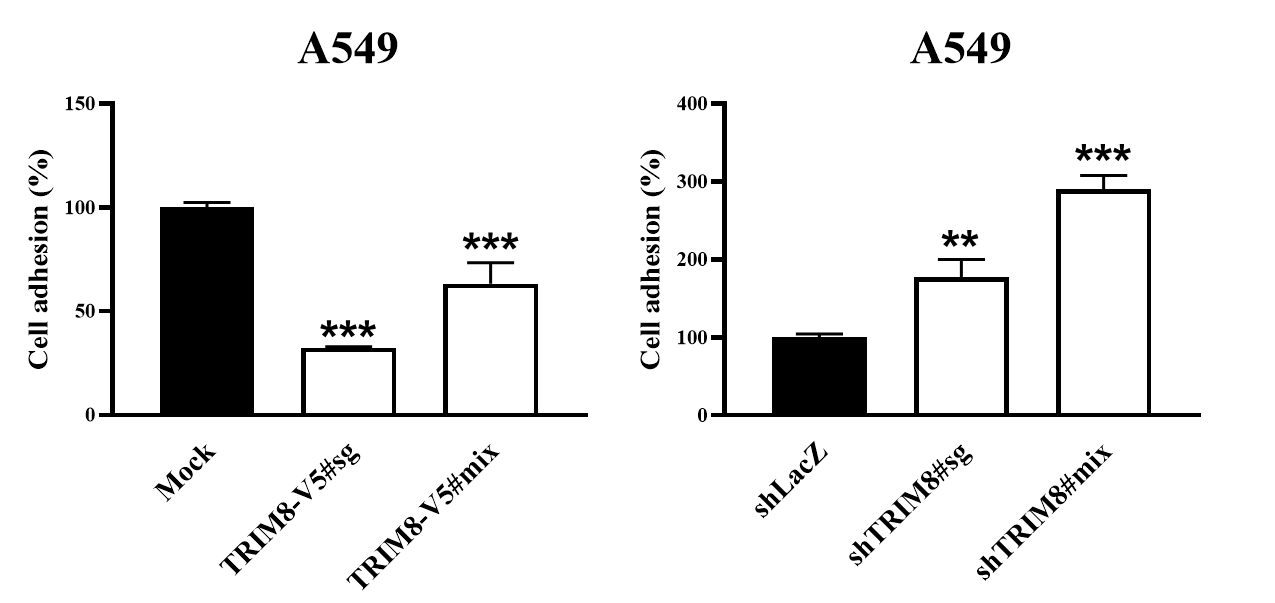
**

**B**

**Figure S5. TRIM8 attenuates the adhesion of A549 lung cancer cells.**

**A** Images of the morphology of mock control, TRIM8-overexpressing, shLacZ control and shTRIM8-knockdown A549 cells. **B** Adhesion of A549 lung cancer cells. TRIM8-V5#sg: single clone with TRIM8 overexpression; TRIM8-V5#mix: mixed clone with TRIM8 overexpression; shTRIM8#sg: single clone with TRIM8 knockdown; shTRIM8#mix: mixed clone with TRIM8 knockdown. The abovementioned data are presented as the means ± SDs of three independent experiments. ***P* < 0.01 and ****P* < 0.001.

**Supplementary Figure S6**

**
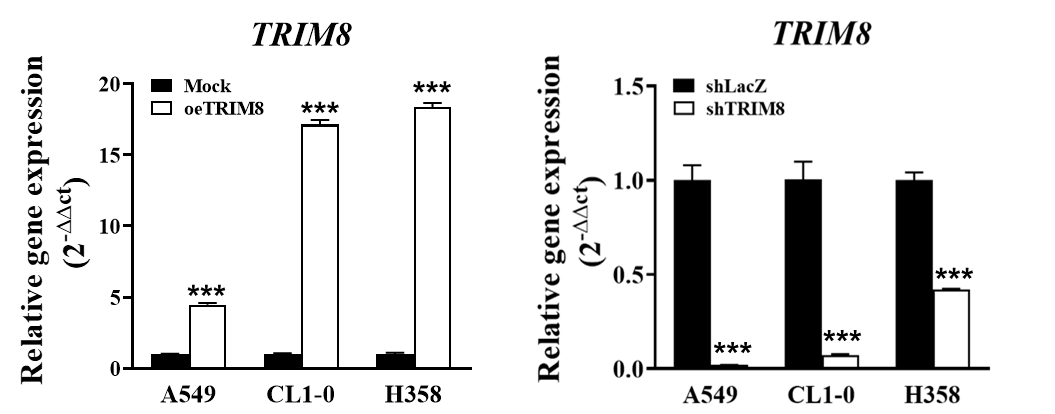
 A**

**
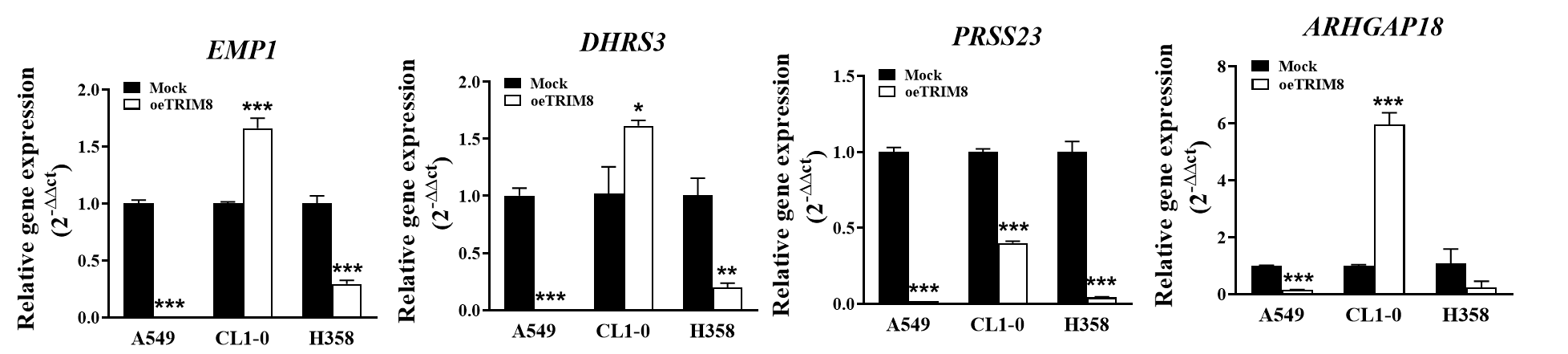
B**

**
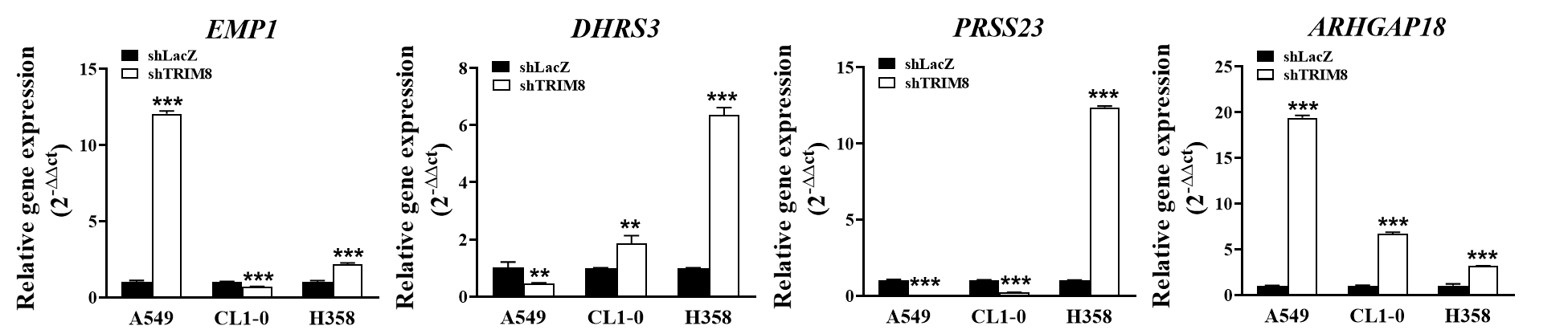
C**

**Figure S6.** TRIM8-regulated gene expression was assessed by RT‒qPCR.

**A** The expression level of TRIM8 in mock control, oeTRIM8, shLacZ control and shTRIM8 transfectants. **B** The expression levels of candidate genes in mock control and oeTRIM8 transfectants were detected via RT‒qPCR. **C** The expression levels of candidate genes in shLacZ control and shTRIM8 transfectants were detected via RT‒qPCR. The abovementioned data are presented as the means ± SDs of three independent experiments. *TBP* was used as an internal control. ***P* < 0.01 and ****P* < 0.001.

**Supplementary Figure S7**

**
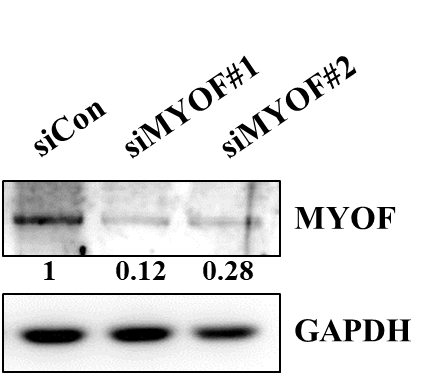

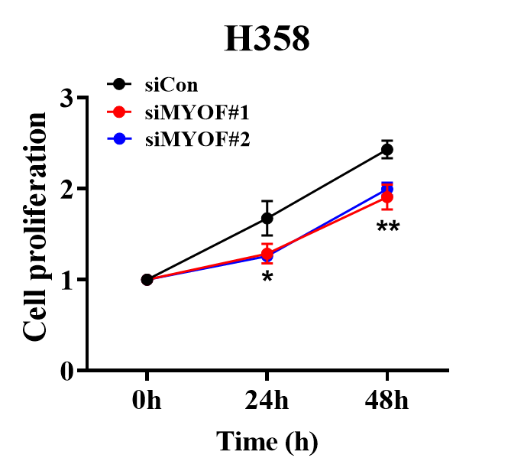
A B**

**
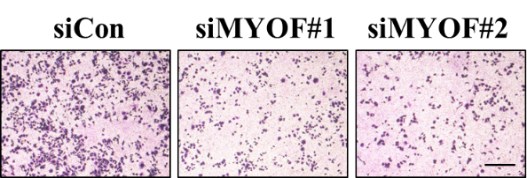
C**

**
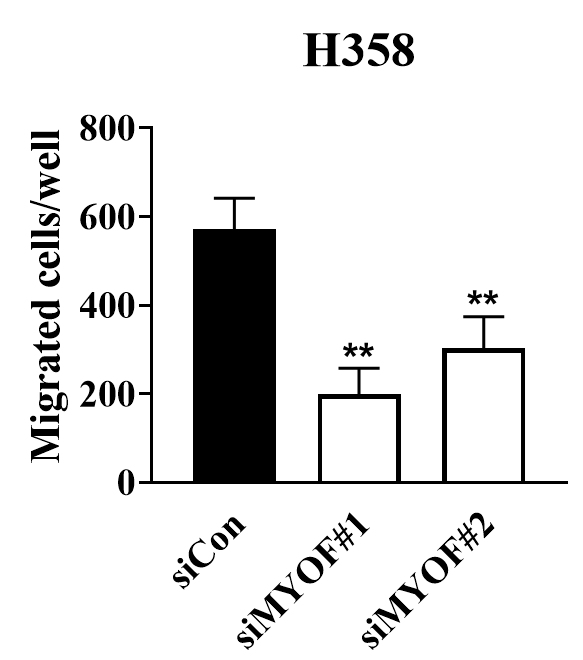
**

**Figure S7. Knockdown of MYOF inhibits the proliferation and migration of H358 cells.**

**A** Transfection of H358 cells with a control siRNA, siMYOF#1 or siMYOF#2. The expression levels of MYOF were detected by western blotting. The MYOF protein levels were quantified using ImageJ, with GAPDH serving as a control. **B** Viability of H358 cells following MYOF silencing, as determined using the PrestoBlue™ reagent. **C** Transwell migration assays were performed to assess the migratory ability of H358 cells following MYOF silencing. Scale bar=50 μm. The abovementioned data are presented as the means ± SDs of three independent experiments. *P < 0.05 and **P < 0.01 compared with the siCon control groups.

**
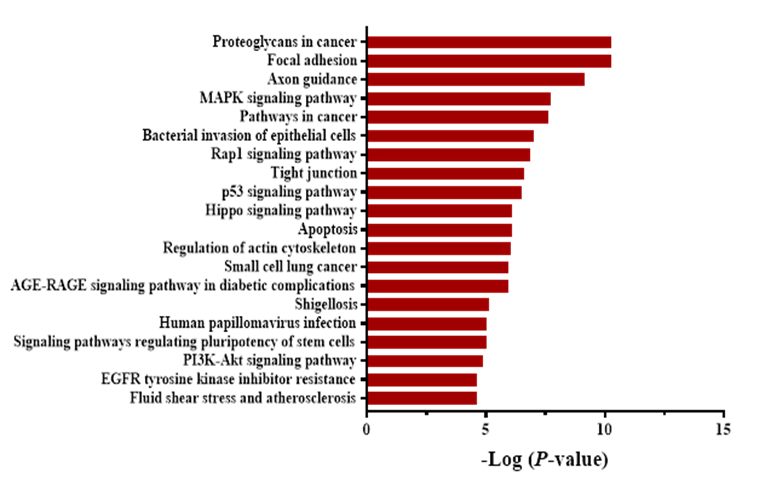
Supplementary Figure S8**

**Figure S8. KEGG pathway enrichment analysis of DEGs.**

KEGG pathway enrichment analysis of DEGs. The vertical axis represents the pathway category, and the horizontal axis represents the −log_10_-transformed p value. The data were analysed using DAVID bioinformatics tools.

**Supplementary Figure S9**

**
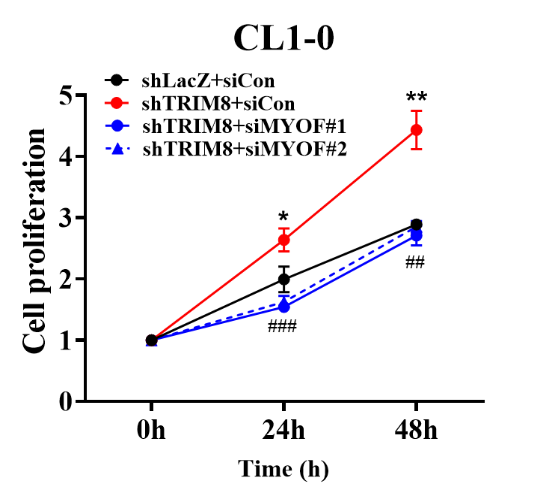

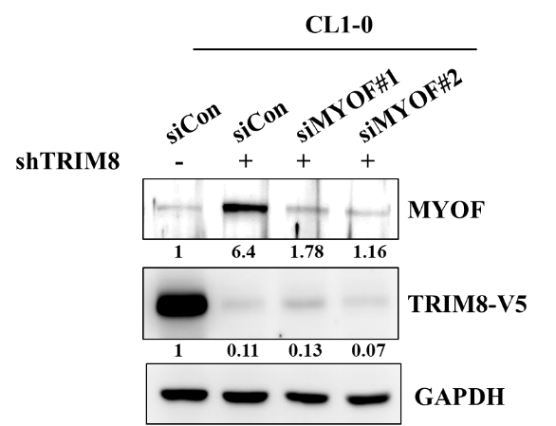
A** **B**

**
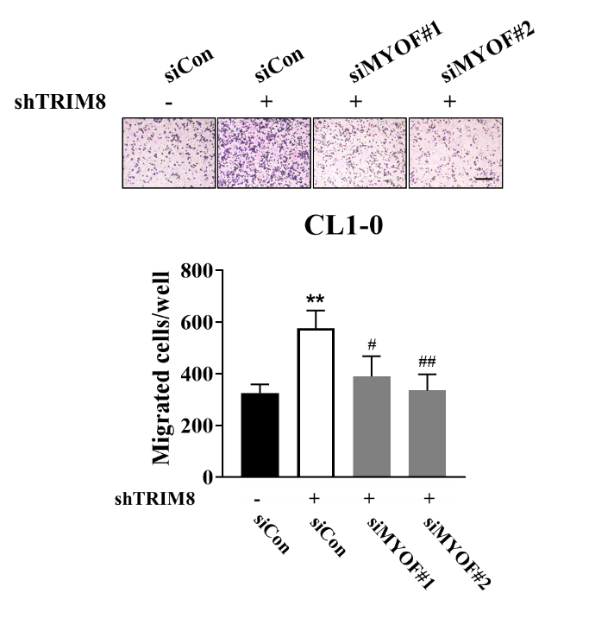
C**

**Figure S9. Silencing of MYOF suppresses the** **increased cell proliferation and migration induced by TRIM8 depletion. A** Transfection of TRIM8-silenced CL1-0 transfectants with a control siRNA, siMYOF#1 or siMYOF#2. The expression levels of TRIM8-V5 and MYOF were detected by western blotting and quantified using ImageJ, with GAPDH serving as a control. **B** Viability of CL1-0 cells with or without double silencing of TRIM8 and MYOF, as determined using the PrestoBlue™ reagent. **C** Transwell migration assays were performed to detect the migration ability of CL1-0 cells with or without double silencing of TRIM8 and MYOF. Scale bar=50 μm. The abovementioned data are presented as the means ± SDs of three independent experiments. **P* < 0.05 and ***P* < 0.01 compared with the shLacZ control groups. ^#^*P* < 0.05, ^##^*P* < 0.01, and ^###^*P* < 0.001 compared with the TRIM8-knockdown groups.

**Supplementary Figure S10**

**
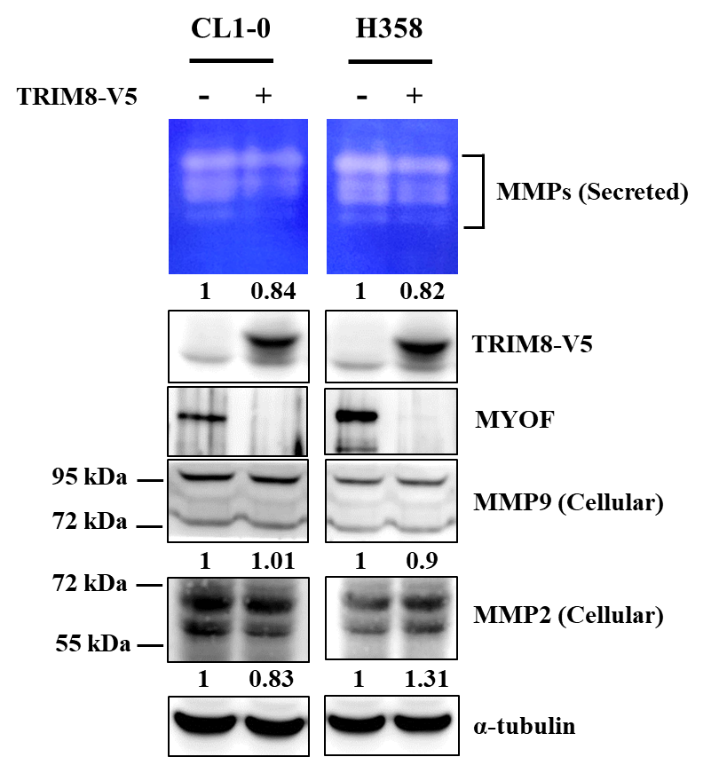
**

**Figure S10. Detection of MMP9 and MMP2 activities by gelatine zymography and western blotting.**

Gelatine zymography was used to detect MMP2 and MMP9 activities. The cellular levels of MMP2, MMP9, TRIM8-V5 and MYOF in mock- and oeTRIM8-transfected cells was confirmed by western blotting. α-Tubulin served as an internal control. The above data were obtained from three independent experiments. The MMP9 and MMP2 protein levels were quantified using ImageJ, with α-tubulin serving as a control.

**Supplementary Figure S11**


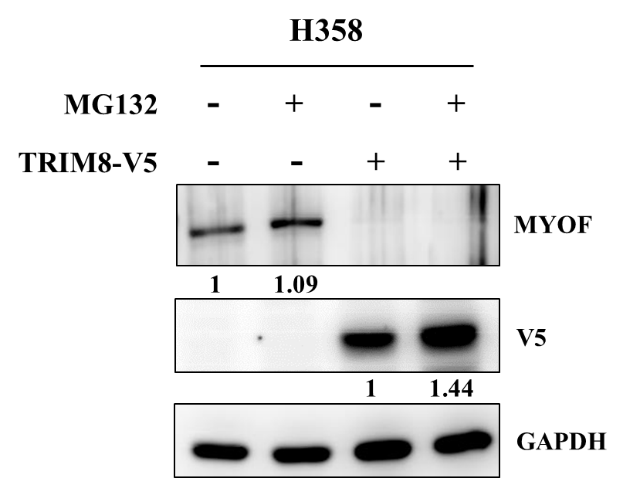

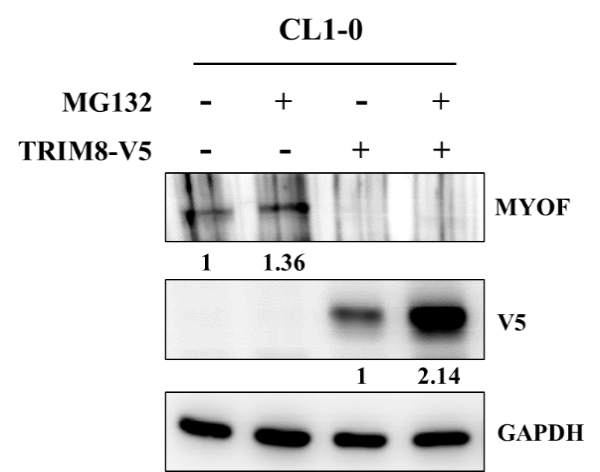
**A B**

**Figure S11. MG132 treatment fails to restore endogenous MYOF expression that was suppressed by TRIM8.**

**A** After 6 hours of treatment with or without MG132, the expression of endogenous MYOF in mock- and oeTRIM8-transfected CL1-0 cells was confirmed by western blotting. **B** After 6 hours of treatment with or without MG132, the expression of endogenous MYOF in mock- and oeTRIM8-transfected H358 cells was confirmed by western blotting. The above data were obtained from three independent experiments. The MYOF and TRIM8-V5 protein levels were quantified using ImageJ, with GAPDH serving as a control.

**Supplementary Figure S12**


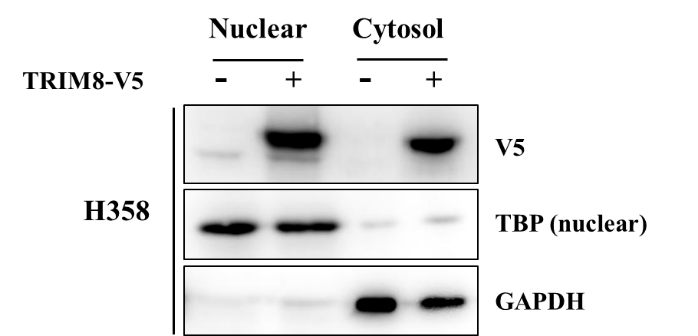
**
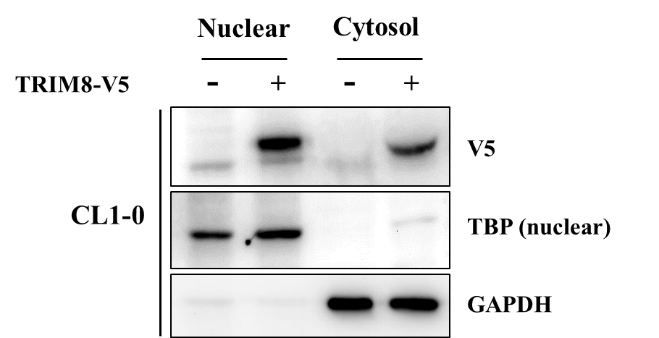
A B**

**Supplementary Figure S12. TRIM8 is located in both the nucleus and the cytoplasm.**

**A** Expression of TRIM8 in the nucleus and cytoplasm of transfected CL1-0 cells. **B** Expression of TRIM8 in the nucleus and cytoplasm of transfected H358 cells. The above data were obtained from three independent experiments. TBP served as an internal control for nuclear expression. GAPDH served as an internal control for cytosolic expression.

**Supplementary Figure S13**

**
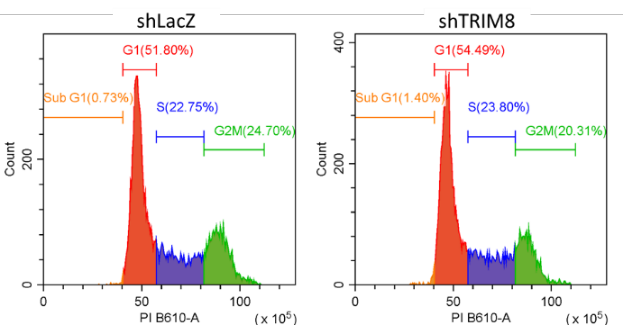
**
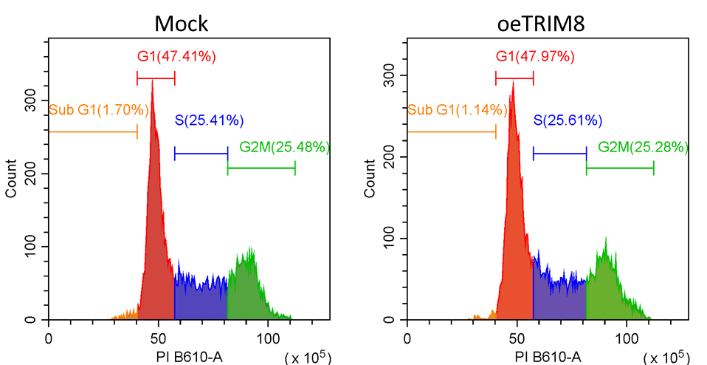
**A B**


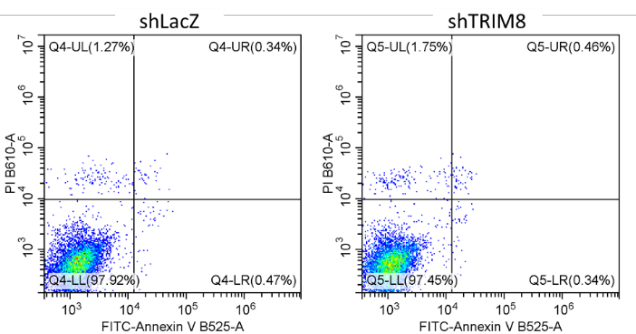

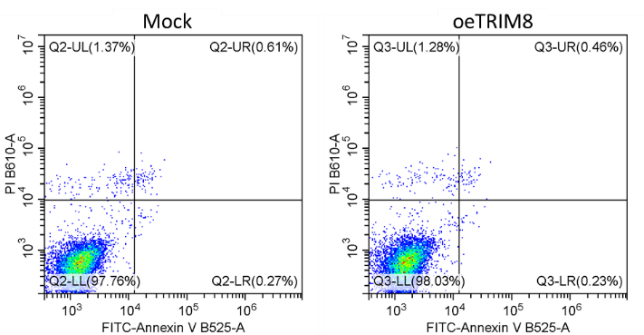
**C D**


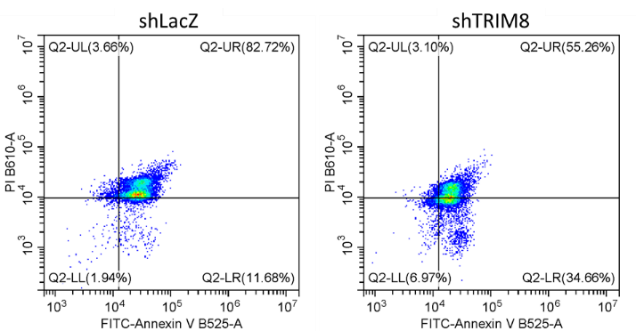

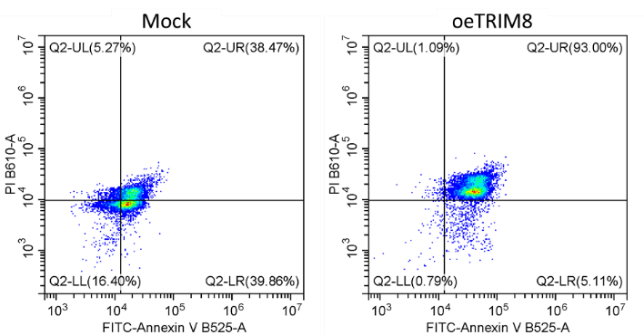
**E F**

**Supplementary Figure S13. Effects of TRIM8 on cell cycle progression and apoptosis in lung cancer cells with or without hydrogen peroxide (H_2_O_2_) exposure.**

**A** and **B** The cell cycle phases of mock, TRIM8-overexpressing, shLacZ control, and TRIM8-silenced lung cancer cells were evaluated using flow cytometry. **C** and **D** Flow cytometry analysis of apoptosis in mock, TRIM8-overexpressing, shLacZ control, and TRIM8-silenced CL1-0 cells. **E** and **F** Mock, TRIM8-overexpressing, shLacZ control, and TRIM8-silenced lung cancer cells were treated with 9.8 µM H_2_O_2_ for 24 h. The cells were analysed using flow cytometry, and the results are presented as the percentages of apoptotic cells.

**Supplementary methods**

**M****acrophage polarization and treatment**

THP-1 cell (ATCC) incubation, differentiation and macrophage polarization were performed according to previous methods (1). THP-1 cells were differentiated into M1 cells via the addition of 1 μg/ml lipopolysaccharide (LPS; Sigma) plus 20 ng/ml interferon-γ (IFN-γ; PeproTech, 300-02), and M2 polarization was induced by treating cells with 20 ng/ml interleukin-4 (IL-4; PeproTech, 200-04). Polarized macrophages were washed and incubated with fresh media for 24 hours, which served as the macrophage subtype-conditioned media, or were directly cocultured with A549 cells in Transwell chambers (0.4-μm pore size filter; Corning Costar, Cambridge, MA, USA). Th1 (IL-1β, IL-6, and IL-12β) or Th2 (IL-10 and CD206) cytokine expression was measured in polarized macrophages to confirm the macrophage subtypes.

**Real-time reverse transcription‒polymerase chain reaction**

Total RNA extracted from cell lines or patient tissues was used for real-time reverse transcription‒polymerase chain reaction (RT‒PCR). The expression of target genes was detected by SYBR Green, and TATA-box binding protein (*TBP*) and *GAPDH* were used to normalize mRNA expression. The experiments were all conducted independently in triplicate. Real-time RT‒PCR was conducted using an ABI StepOnePlusTM Real-Time PCR system and analysed with StepOne software (Applied Biosystems, Life Technologies Corp., Carlsbad, CA, USA). The primer sets used in this study are listed in Additional file 1: Table S1.

**Plasmid and small-interfering RNA (siRNA) transfection and lentiviral infection**

The full-length cDNA of human TRIM8 was cloned and inserted into the pcDNA3.1-V5-His TOPO vector (Thermo Fisher Scientific). pCDNA3.1-MYOF-HA (Plasmid #22443), pRK5-HA-Ubiquitin-K48 (Plasmid #17605) and pRK5-HA-Ubiquitin-K63 (Plasmid #17606) were purchased from Addgene. The plasmids were transfected into A549, CL1-0 and H358 cells with Lipofectamine TM-2000, and geneticin (G418; Merck, Darmstadt, Germany) was subsequently used to select stable cell clones. The TRIM8 shRNA lentiviral vector (TRCN370605) and lacZ control shRNA (pLKO.1-shLacZ, TRCN0000072224) lentiviral vectors were obtained from the National RNAi Core Facility (Academia Sinica, Taiwan). The TRIM8-overexpressing cells were infected with the lentivirus in medium containing polybrene (8 μg/ml). After infection for 24 h, the cells were treated with 2.5 μg/ml puromycin for 3–7 days. Transfection of the siRNAs was performed using Lipofectamine RNAiMAX reagent according to the manufacturer's protocol (Invitrogen). The final concentration of each siRNA was 30 nM. Human TRIM8 siRNA (#sc-90801), MYOF siRNA (#sc-72293) and control siRNA (#sc-37007) were purchased from Santa Cruz Biotechnology, the TRIM8 and MYOF siRNA containing three distinct sequences. The sequences of the other siRNAs used are as follows: siTRIM8#1 (sense: GCGGAGAAUUGGAAGAACUTT; antisense: AGUUCUUCCAAUUCUCCGCTT), siTRIM8#2 (sense: GGAGAUCCGAAGGAAUGAATT; antisense: UUCAUUCCUUCGGAUCUCCTT), siMYOF#1 (sense: GGCGGAUGCUGUCAAAUAATT; antisense: UUAUUUGACAGCAUCCGCCTT), and siMYOF#2 (sense: GGGACAUCGUUAUCGAAAUTT; antisense: AUUUCGAUAACGAUGUCCCTT).

**Western blot analysis**

The cells were lysed with RIPA lysis buffer (50 mM Tris-HCl, 150 mM NaCl, 1 mM EDTA, and 1% NP-40). Western blot procedures were performed as previously described (2). Primary antibodies against the V5 tag (Thermo Fisher Scientific, R960-25), MYOF (Santa Cruz Biotechnology, sc-376879), MMP2 (Santa Cruz Biotechnology, sc-10736), MMP9 (Cell Signaling Technology, #3852), ubiquitin (Santa Cruz Biotechnology, sc-166553), HA tag (Santa Cruz Biotechnology, sc-7392) and TRIM8 (Santa Cruz Biotechnology, sc-398878) were diluted from 1:1000 to 1:5000. The antibodies against GAPDH (Santa Cruz Biotechnology, sc-32233) or α-tubulin (GeneTex, GTX628802) were used as internal controls.

**Cell proliferation and clonogenic assay**

Human NSCLC cell lines were seeded in 96-well plates at a density of 4000 cells/well and cultured for 16 h. PrestoBlue™ reagent (Invitrogen) was added at 0, 24, 48 and 72 h to monitor cell viability. The absorbance was measured at 570 nm using a Victor3 spectrophotometer (Perkin-Elmer, Santa Clara, CA, USA). For the anchorage-dependent colony formation assay, 500 cells were seeded in six-well plates and cultured for 7 to 9 days. The colonies were fixed with 4% paraformaldehyde and stained with 0.05% crystal violet. The images were analysed using ImageJ software (National Institutes of Health, Bethesda, MD, USA). The colony formation ability was evaluated according to the area of ​​the well covered by the colonies.

**Cell attachment assay**

The attachment assay using the colorimetric detection of bound cells was performed as described previously (3). Cells were seeded into a 96-well plate at a density of 1×10^6^ cells/ml in 100 μl of medium per well and cultured for 30 minutes. The cells in the 96-well plate were fixed and stained with 100 µL of 0.1% crystal violet in 4% paraformaldehyde at 4°C overnight. After the nonadherent cells were removed by washes with 1× PBS, 100 μl of 10% acetic acid was added to each well to solubilize the attached cells. The absorbance was measured at 570 nm using a Victor3 spectrophotometer.

**Migration and invasion assays**

A wound healing assay (2) was performed to analyse horizontal cell mobility, and the percentage of the cell recovery area was measured and compared with the value calculated at 0 h. The upper chambers of Transwells (8-μm pore size filter; Corning Costar, Cambridge, MA, USA) were seeded with 5 × 10^3^ cells to measure vertical cell mobility. The upper chamber of a Transwell filter coated with Matrigel (BD Bioscience) was seeded with 1 × 10^5^ cells to analyse cell invasion. For vertical migration and invasion tests, medium containing 10% FBS medium was added to the lower chambers and the plates were incubated for 24 hours. The cells that migrated to the lower surface of the polycarbonate filter were fixed, stained, and counted under a light microscope.

**Gelatine zymography assay**

The procedures were performed as previously described, with some modifications (4). Briefly, the collected conditioned media were concentrated with Amicon^®^ Ultra0.5 filters (Merck, UFC501024). Protein (0.2 μg) from each sample was loaded in wells and separated on SDS‒PAGE gels containing 4 mg/mL gelatine (Sigma‒Aldrich). The gels were incubated with incubation buffer at 37°C for 16 hours, stained with staining solution for 15 minutes, and then destained with H_2_O until the bands were visible.

**Coimmunoprecipitation assay**

The cells were lysed with cell lysis buffer (50 mM Tris-HCl, 150 mM NaCl and 0.1% NP-40). The indicated antibodies were added to the cell lysate, which was subsequently incubated overnight at 4°C. Then, Protein A magnetic beads (Invitrogen) were added and incubated for 4 hours at 4°C. The magnetic beads were then washed three times with cold lysis buffer, and the Co-IP products were analysed via western blotting.

**Cell cycle analysis**

Stable CL1-0 clones were seeded at a density of 2×10^5^ cells/well in 6-well plates. The cells were harvested with trypsin and subsequently fixed with 75% ice-cold ethanol at -20°C for a minimum of one hour. Next, 0.5 ml of 0.5% Triton X-100 in PBS and RNase (1 mg/ml) were added to the sample, which was subsequently incubated at 37°C for 30 minutes. Finally, 0.5 ml of propidium iodide (50 μg/ml) was added, and the mixture was incubated at room temperature for 10 minutes. The cell cycle (G1/G0, S and G2/M phases) was detected with an FC500 flow cytometer (Beckman Coulter Inc., Brea, CA, USA).

**Apoptosis assay**

An Annexin V-FITC Apoptosis Detection Kit (BD Pharmingen, San Jose, CA, USA) was used to assess apoptosis. The cells were cultured in 6 cm dishes with or without 9.8 µM hydrogen peroxide (H₂O₂) for 24 hours. The cell pellets were washed with PBS and resuspended in 1x binding buffer. The cells were subsequently stained with Annexin V-FITC (fluorescein isothiocyanate) followed by propidium iodide. The percentages of viable (FITC-negative and PI-negative), early apoptotic (FITC-positive and PI-negative), late apoptotic (FITC-positive and PI-positive), and necrotic (FITC-negative and PI-positive) cells were detected with a Beckman Coulter FC500 flow cytometer and CytoFLEX.

**References**

1. Yuan A, Hsiao YJ, Chen HY, Chen HW, Ho CC, Chen YY, et al. Opposite Effects of M1 and M2 Macrophage Subtypes on Lung Cancer Progression. Sci Rep. 2015;5:14273.

2. Tsai MF, Wang CC, Chang GC, Chen CY, Chen HY, Cheng CL, et al. A new tumor suppressor DnaJ-like heat shock protein, HLJ1, and survival of patients with non-small-cell lung carcinoma. J Natl Cancer Inst. 2006;98(12):825-38.

3. Humphries MJ. Cell adhesion assays. Methods Mol Biol. 2009;522:203-10.

4. Chu YW, Yang PC, Yang SC, Shyu YC, Hendrix MJ, Wu R, et al. Selection of invasive and metastatic subpopulations from a human lung adenocarcinoma cell line. Am J Respir Cell Mol Biol. 1997;17(3):353-60.
