# Supplementary material for: E3 ligase TRIM8 suppresses lung cancer metastasis by targeting MYOF degradation through K48-linked polyubiquitination
Source: Cell Death Dis. 2025 Feb 11;16(1):88. doi: 10.1038/s41419-025-07421-6 (PMC11814372; doi:10.1038/s41419-025-07421-6)

Figure 1D

TRIM8

GAPDH

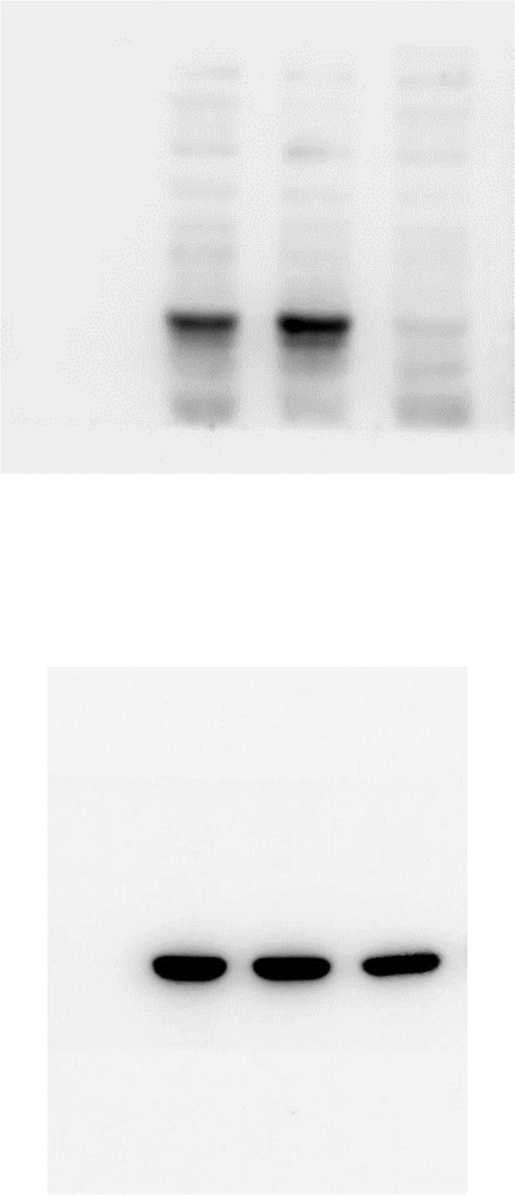

Figure 3A

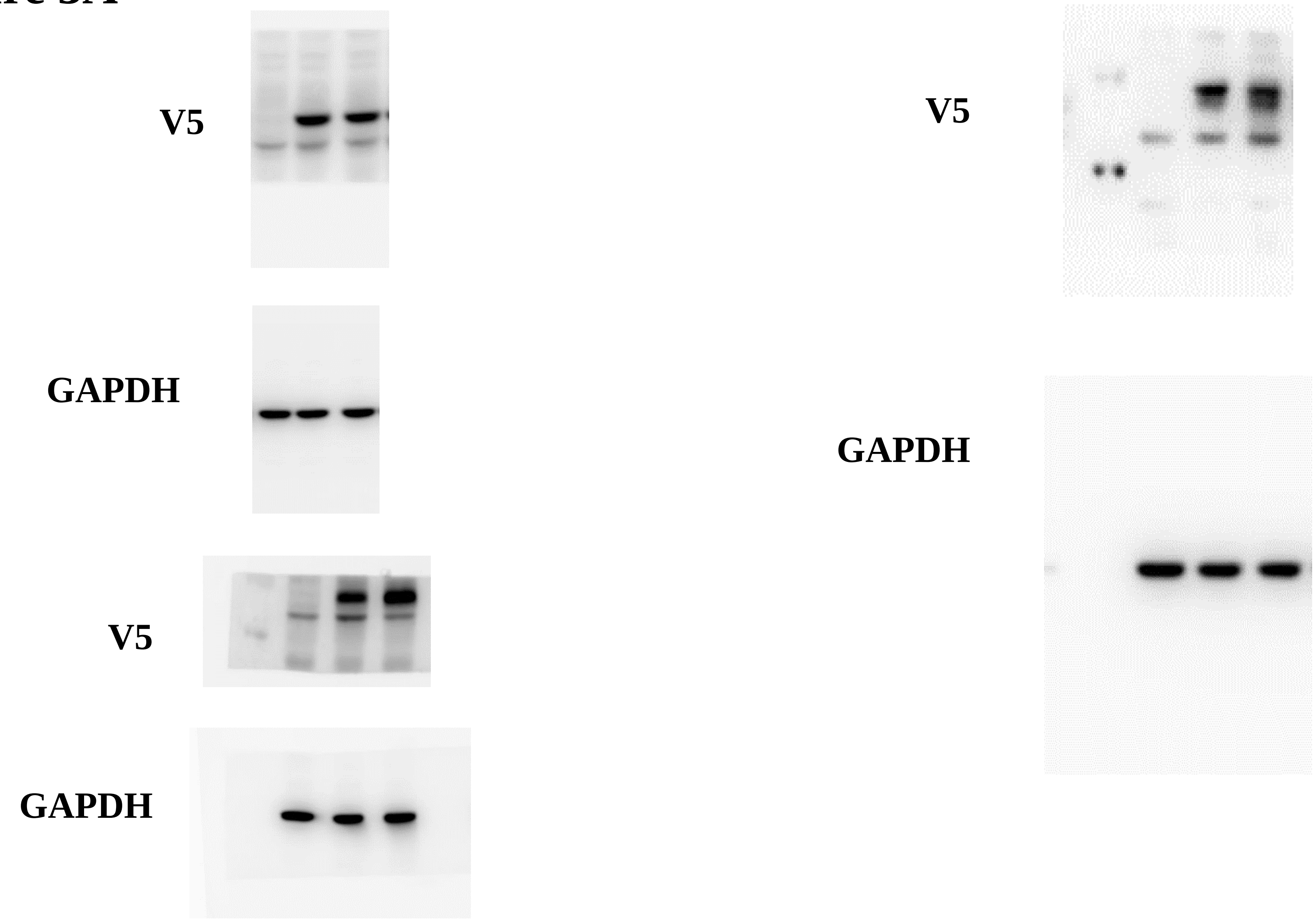

Figure 3B

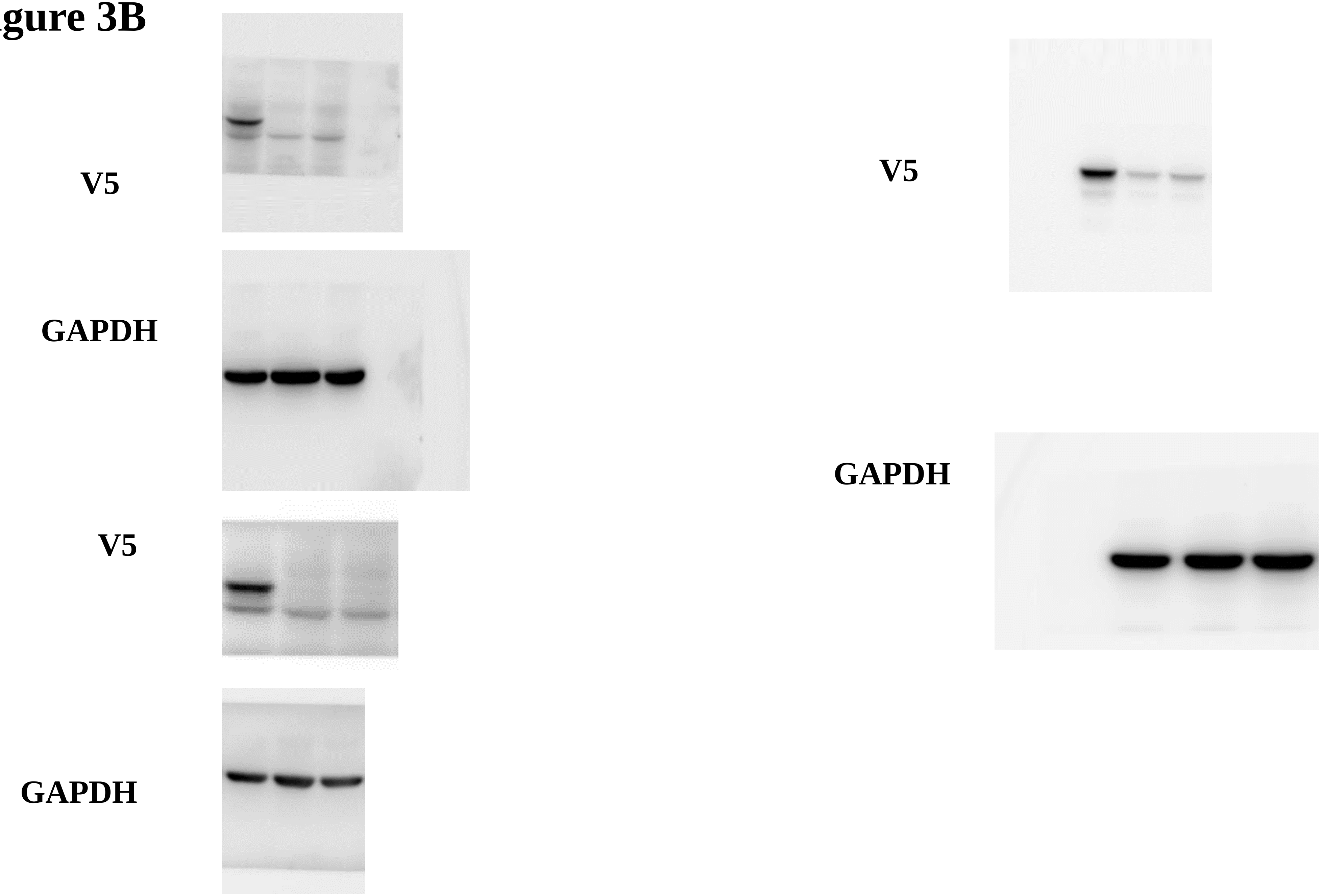

Figure 6E

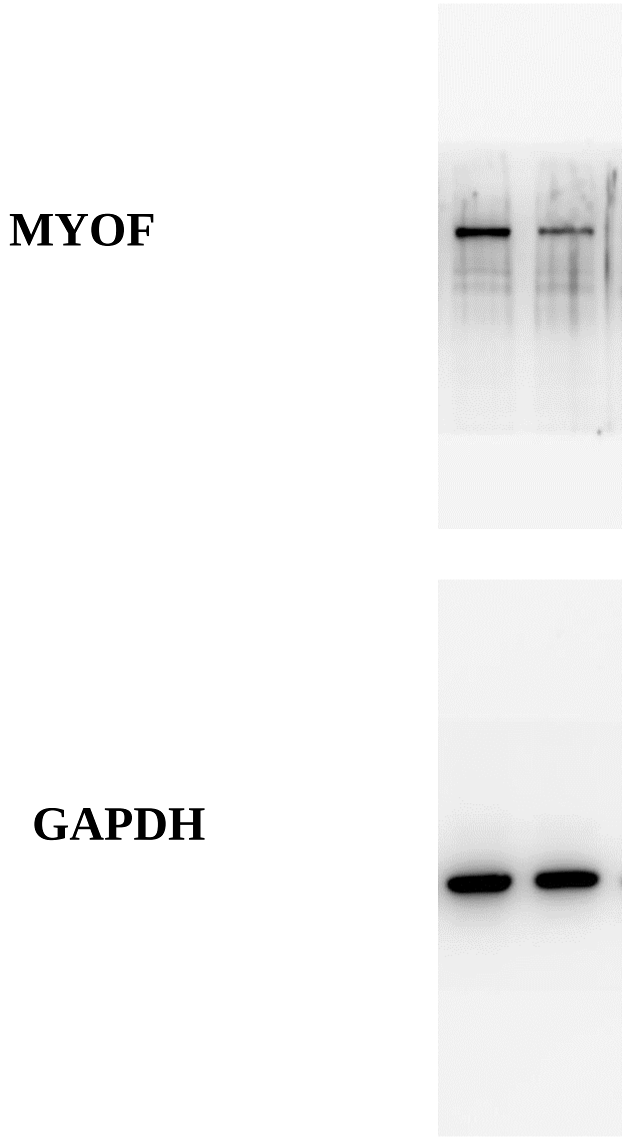

Figure 7A

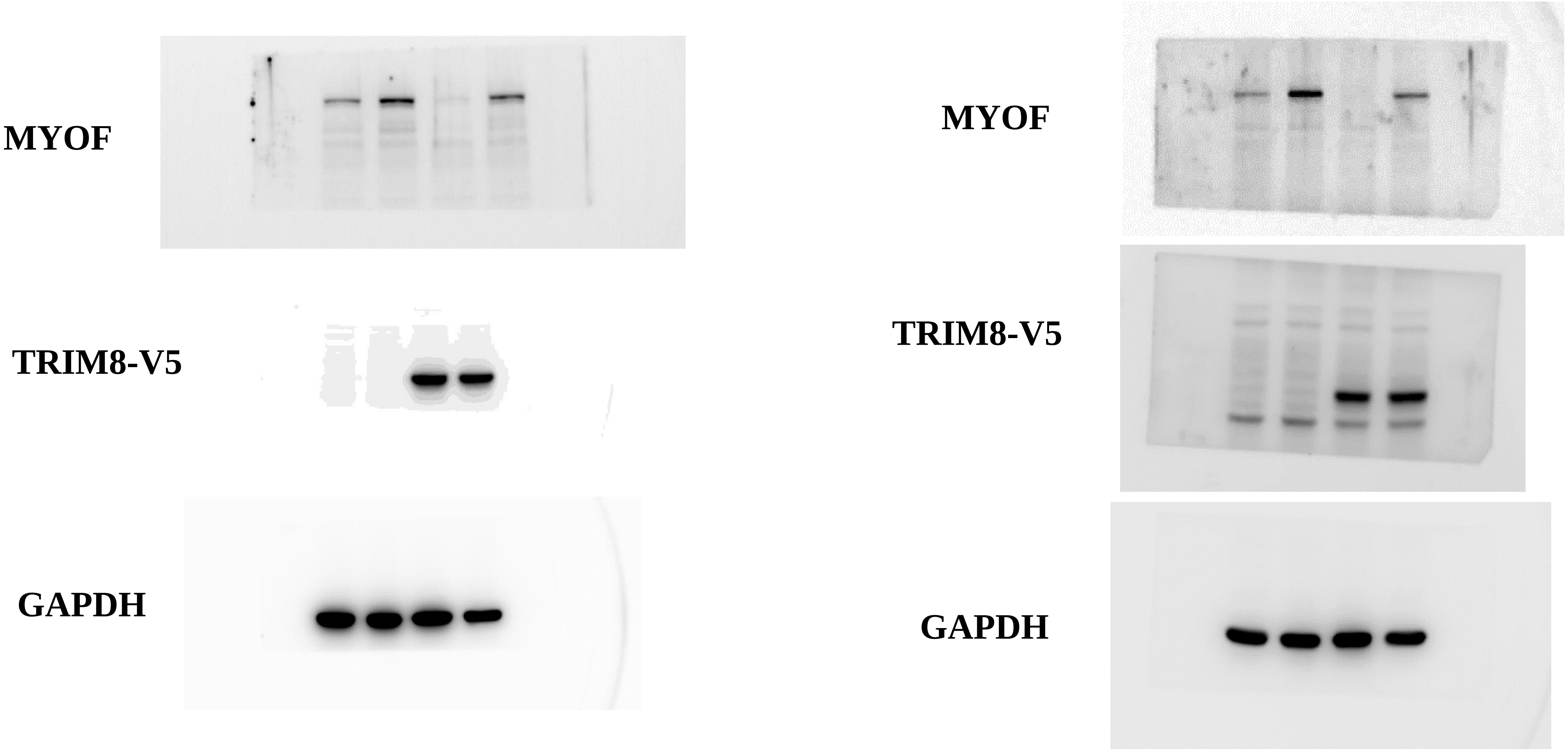

Figure 7F

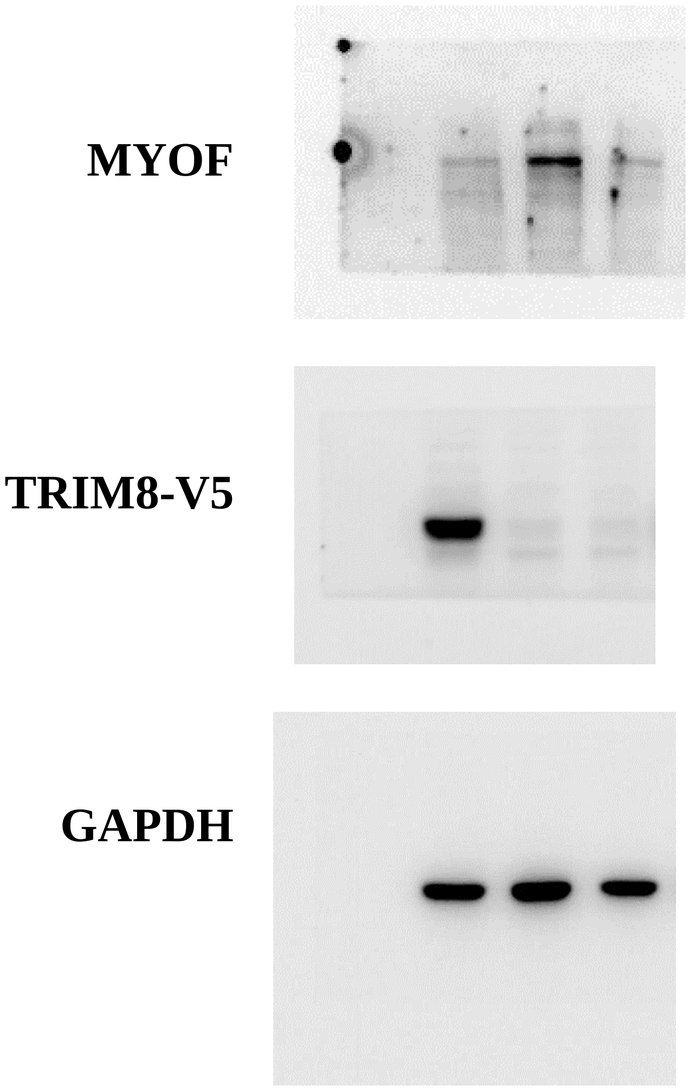

Figure 8A and B

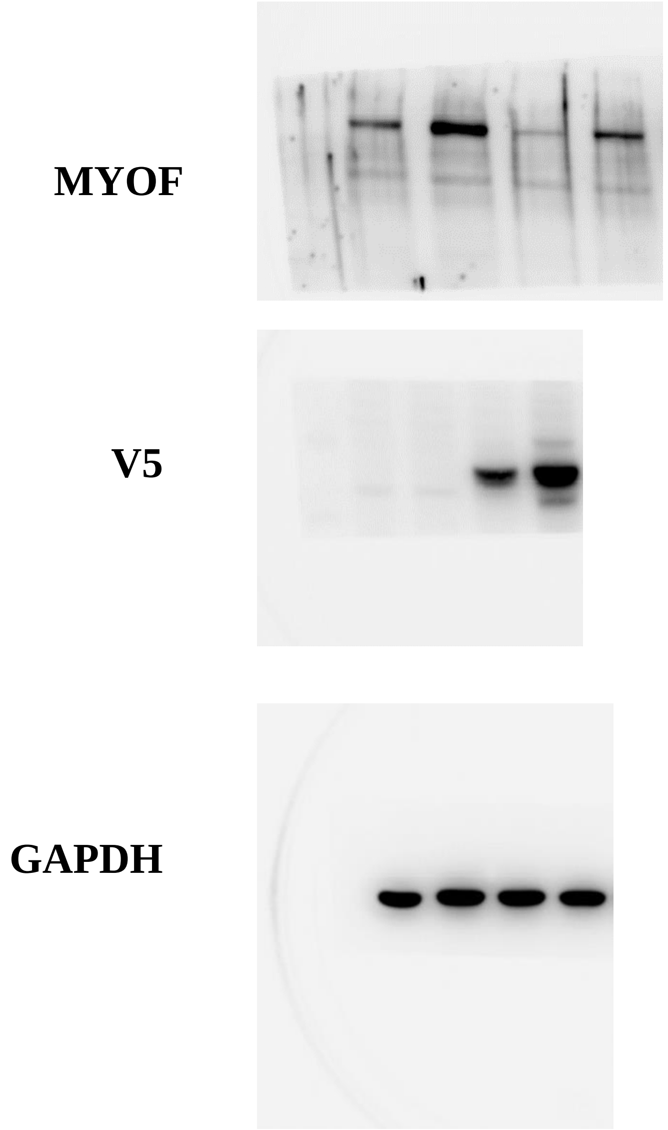

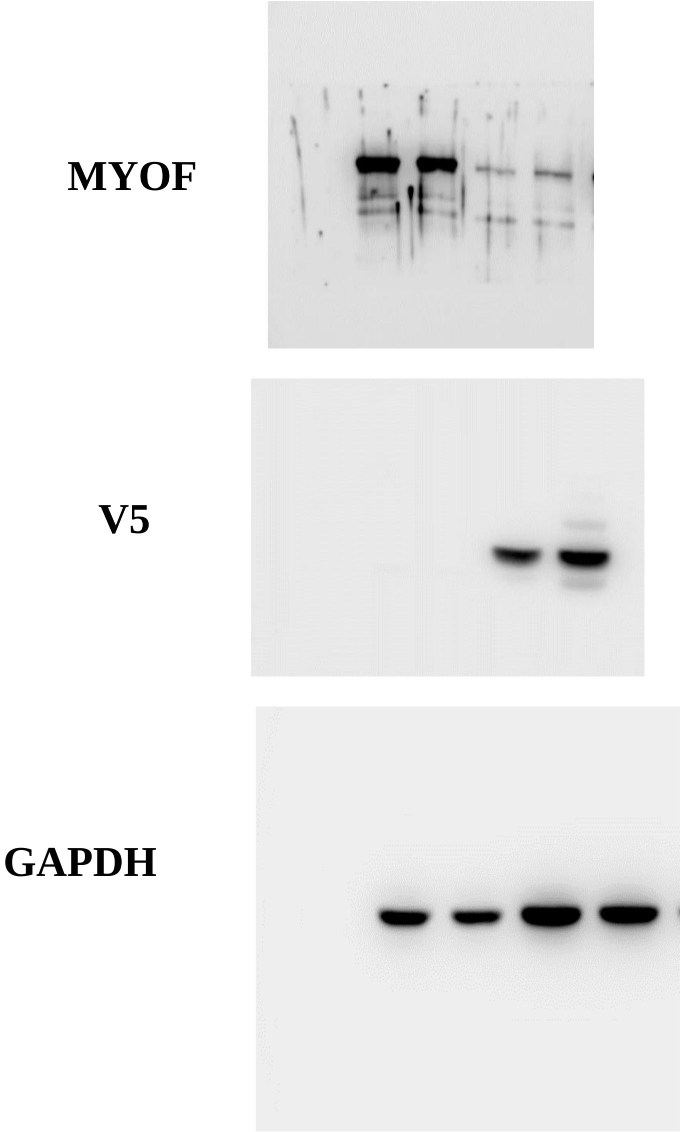

Figure 8C

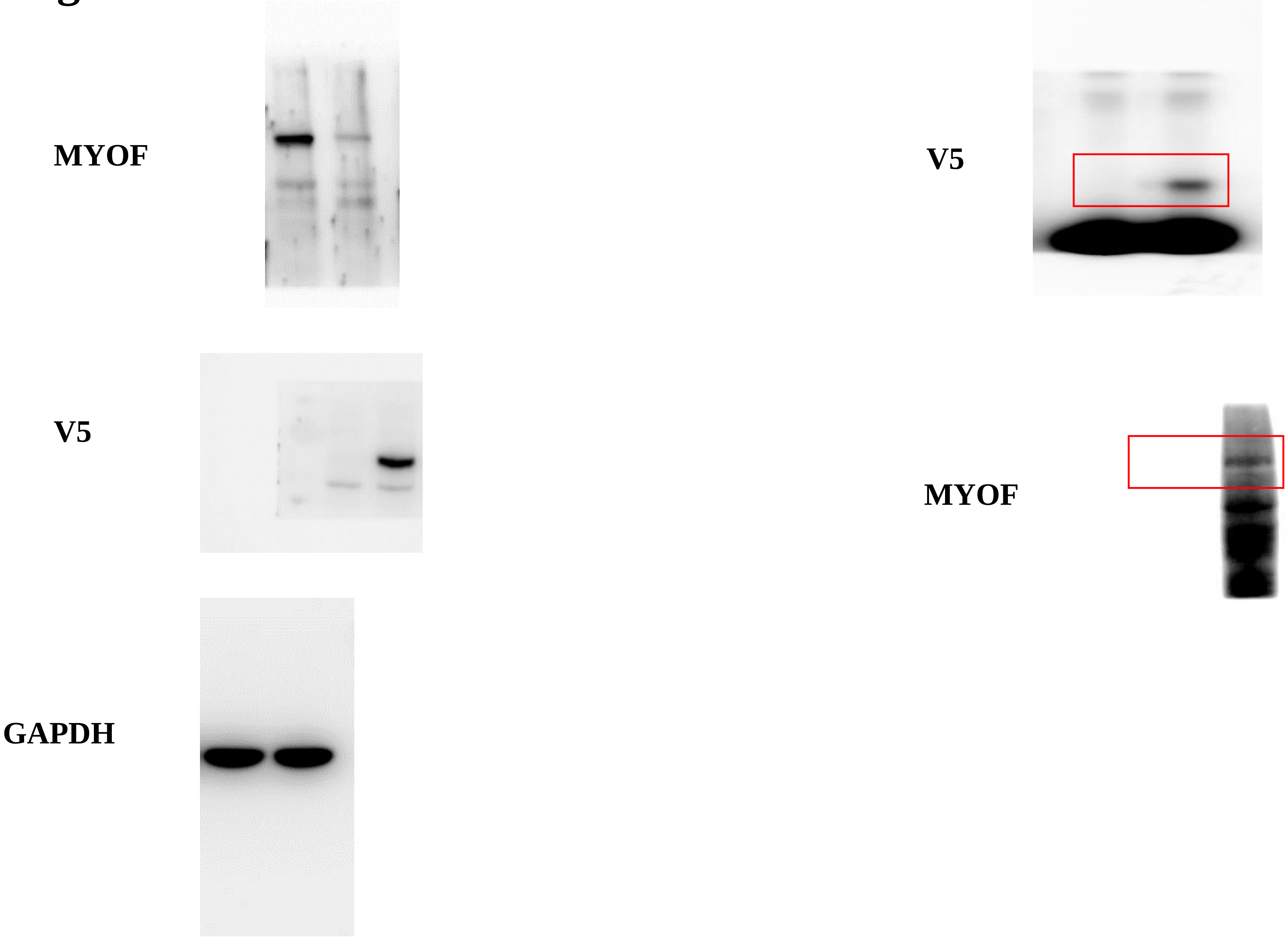

Figure 8D

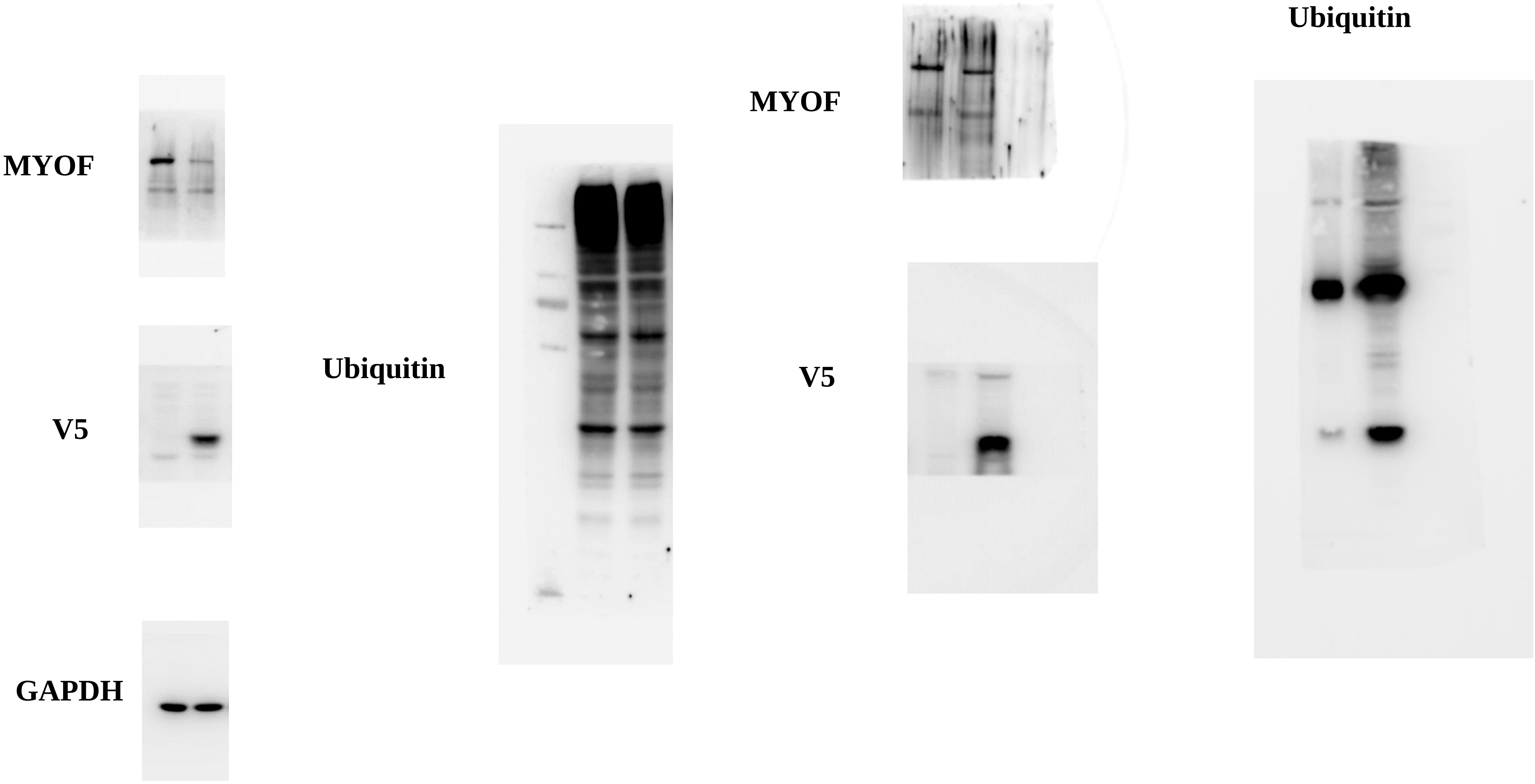

Figure 8E (left panel)

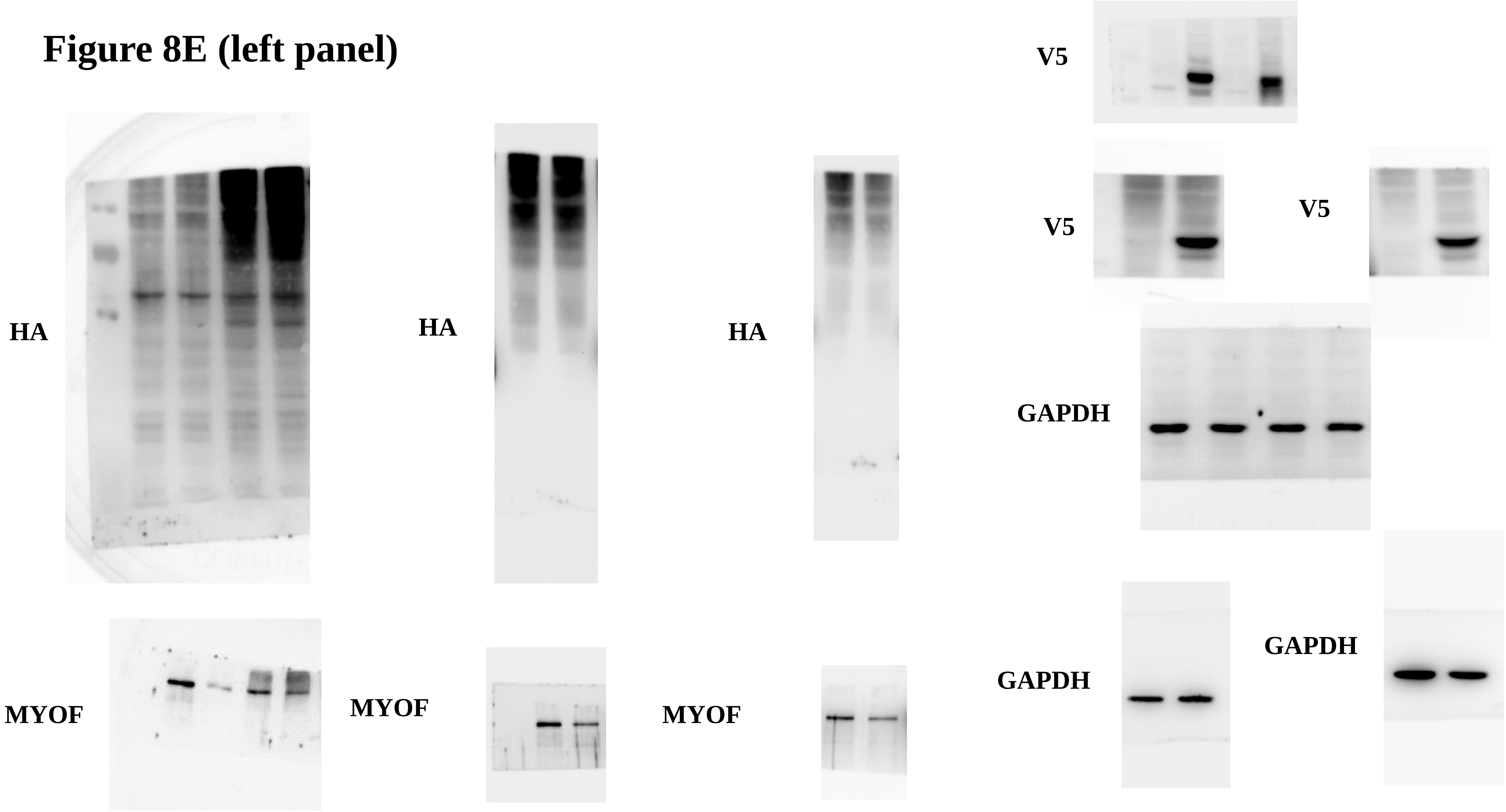

Figure 8E (right panel)

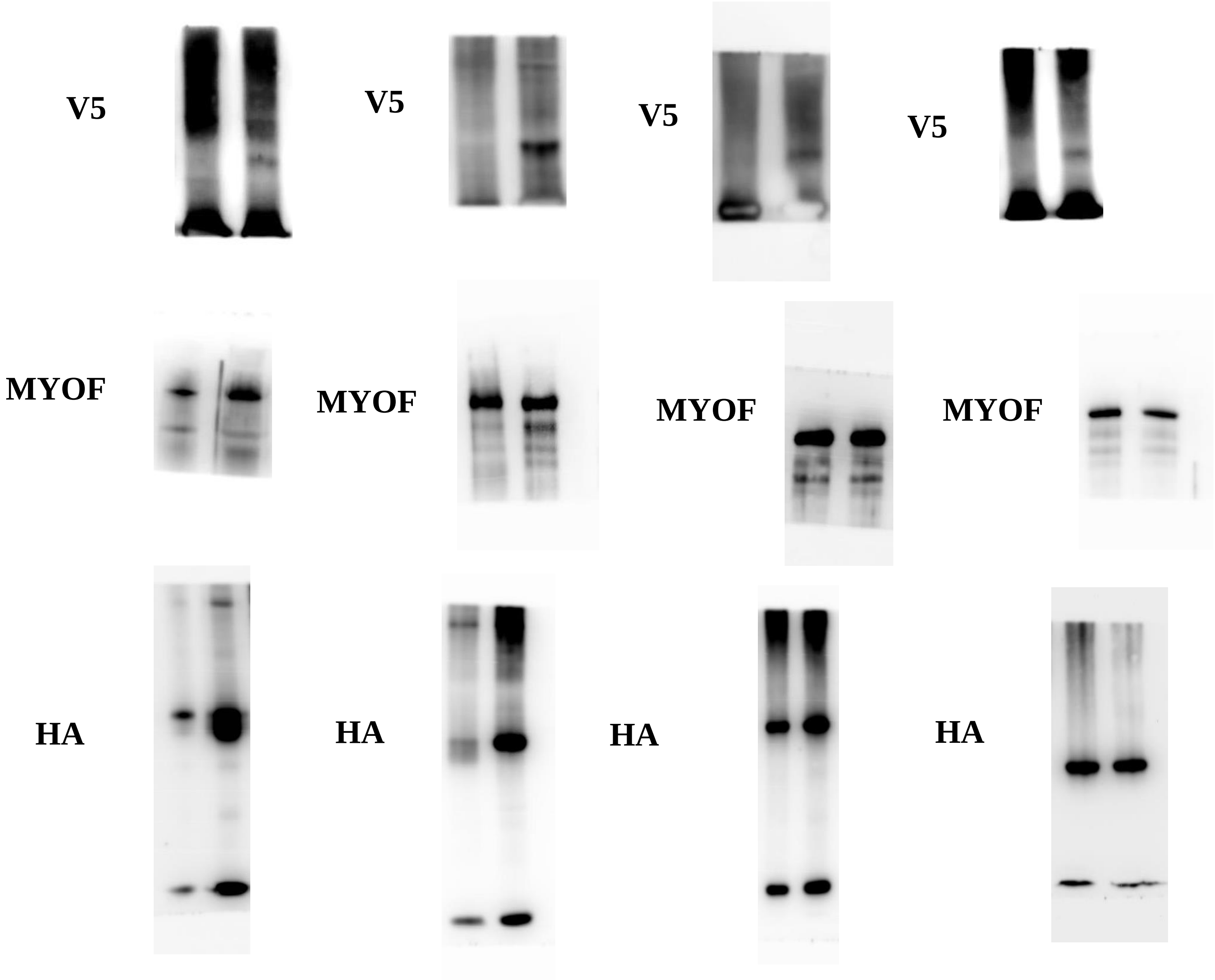

Supplementary Figure S3

TRIM8

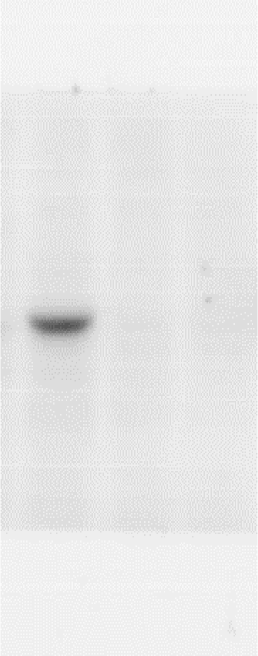

GAPDH

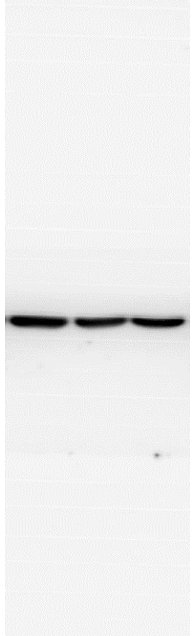

Supplementary Figure S7

MYOF

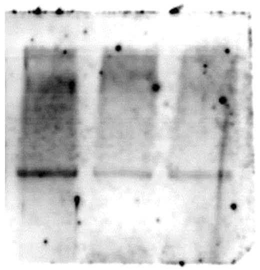

GAPDH

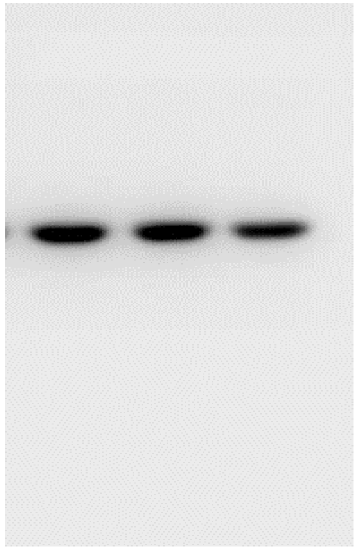

Supplementary Figure S9

MYOF

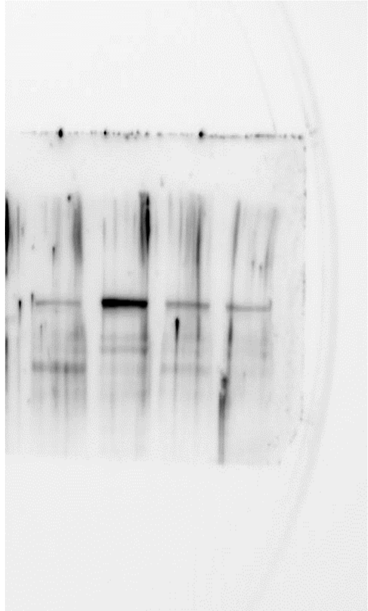

GAPDH

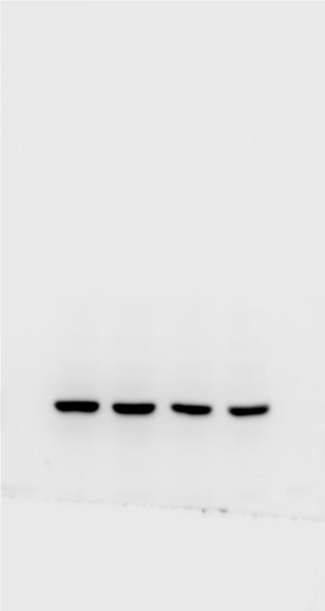

TRIM8-V5

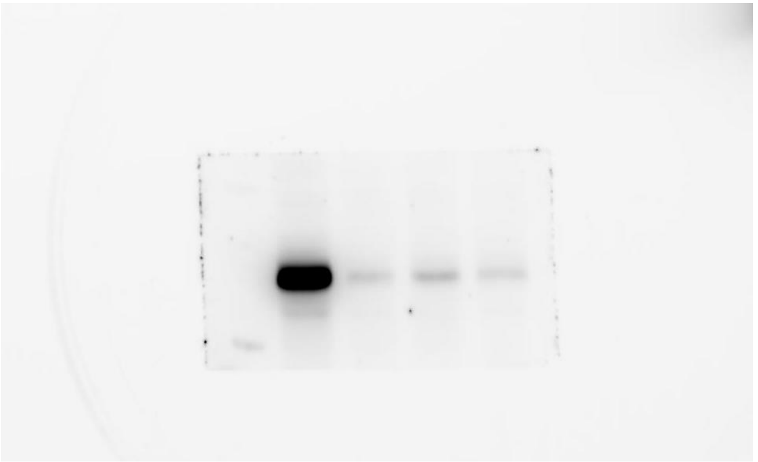

Supplementary Figure S10

TRIM8-V5

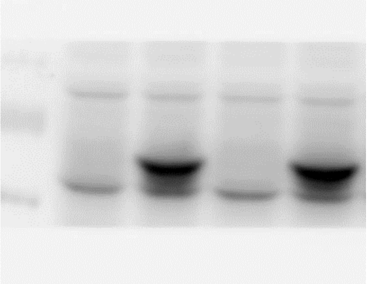

MYOF

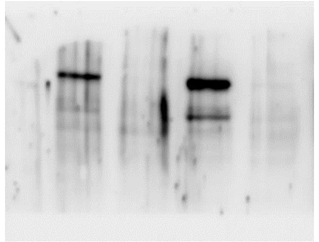

MMP9 (Cellular)

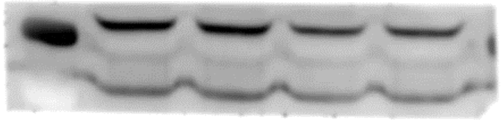

MMP2 (Cellular)

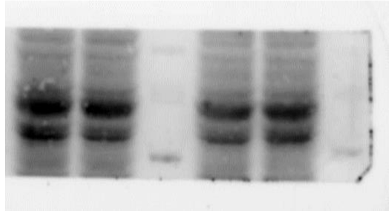

α-tubulin

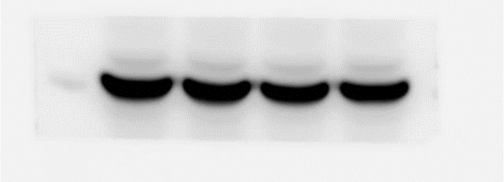

Supplementary Figure S11

MYOF

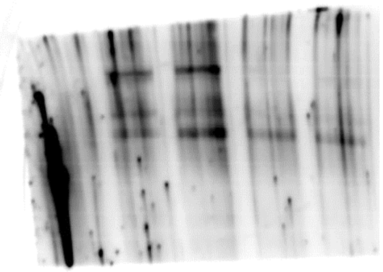

V5

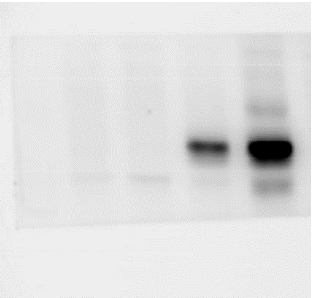

GAPDH

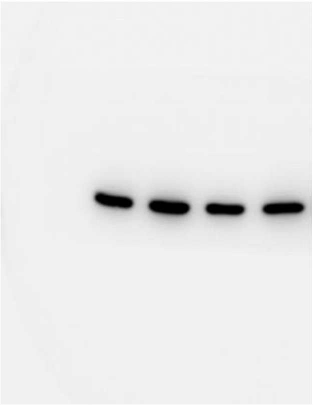

MYOF

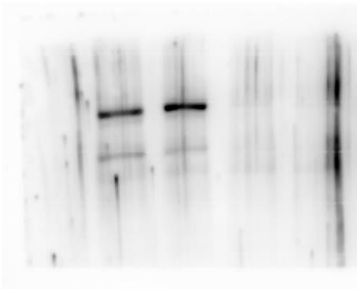

V5

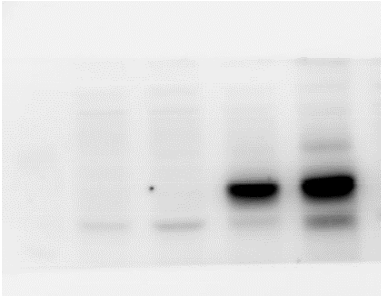

GAPDH

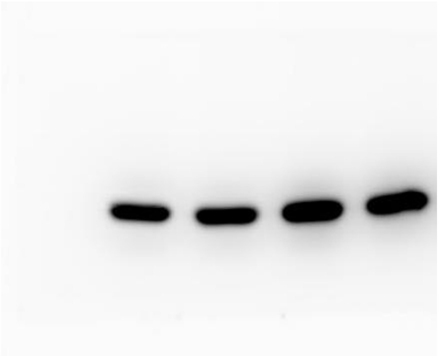

Supplementary Figure S12

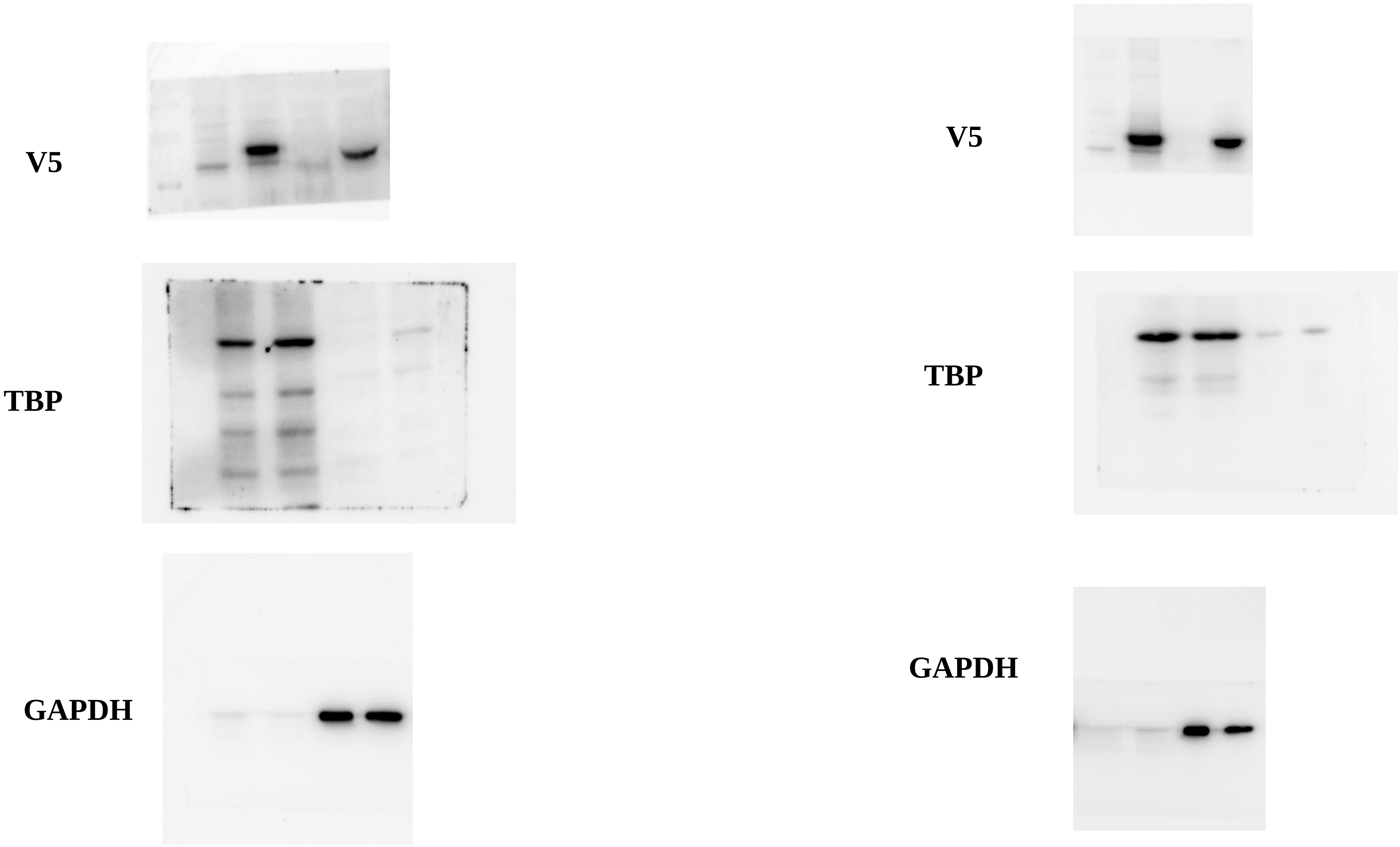

Supplement: Supplementary file 2 — Raw data of western blot [file 41419_2025_7421_MOESM2_ESM.pdf]
